# Adverse drug events associated with insulin glargine: a real-world pharmacovigilance study based on the FAERS database

**DOI:** 10.3389/fphar.2025.1563238

**Published:** 2025-04-28

**Authors:** Tongtong Wang, Gefei He, Wan Xiong, Juanjuan Huang

**Affiliations:** ^1^ Department of Pharmacy, The Affiliated Changsha Hospital of Xiangya School of Medicine, Central South University, Changsha, Hunan, China; ^2^ Department of Pharmacy, The First Hospital of Changsha, Changsha, Hunan, China

**Keywords:** insulin glargine, adverse drug event, FAERS, pharmacovigilance, diabetes mellitus

## Abstract

**Background:**

Insulin glargine is a long-acting drug and the first synthetic insulin to mimic human metabolism. The safety of insulin glargine in the real world remains to be further investigated. This study aims to analyze insulin glargine-related adverse events (ADEs) to guide its safe clinical use.

**Methods:**

This study collected ADE reports from the FDA Adverse Event Reporting System (FAERS) between the first quarter of 2004 and the third quarter of 2024, where insulin glargine was identified as the primary suspect drug. Four disproportionate analytical methods were employed to analyze positive signals for drug-related ADEs, including the Reporting Odds Ratio (ROR), Proportional Reporting Ratio (PRR), Bayesian Confidence Propagation Neural Network (BCPNN), and Multi-item Gamma Poisson Shrinker (MGPS). The study also describes the time to onset of ADEs and uses the Weibull distribution to analyze the temporal trend of ADEs occurrence over time.

**Results:**

This study included 97,350 ADE reports, containing 228,258 ADEs, and identified 130 ADEs with positive signal. The study confirmed several known ADEs, such as hypoglycemia, injection site pain and acquired lipodystrophy. Additionally, several unexpected ADEs were identified, including pancreatic neoplasm, medullary thyroid cancer, and bone marrow tumor cell infiltration. 28.13% of ADEs occurred within the first month. The Weibull distribution indicated that the occurrence of ADEs decreased over time.

**Conclusion:**

This study explored the real-world safety of insulin glargine and revealed several unexpected ADEs. These findings provide new insights into the safety profile of insulin glargine for clinicians.”

## 1 Introduction

Diabetes mellitus is a metabolic disorder characterized by persistent hyperglycemia resulting from impaired insulin secretion, insulin function, or a combination of both (Darenskaya et al., 2021). Researchers estimated that in 2021, the global prevalence of diabetes among individuals aged 20–79 was approximately 10.5% (536.6 million individuals), and it is projected to increase to 12.2% (783.2 million individuals) by 2045 (Sun et al., 2022).

Insulin glargine is a long-acting insulin analog designed to mimic human metabolism (Zhou et al., 2019). It was approved by the U.S. Food and Drug Administration (FDA) in April 2000 for the treatment of diabetes mellitus (Goykhman et al., 2009). Multiple randomized controlled trials (RCTs) have demonstrated that insulin glargine exerts stable and sustained glucose-lowering effects by binding to insulin receptors, promoting glucose uptake and utilization in peripheral tissues, and inhibiting hepatic glucose output. Despite its significant efficacy in glycemic management, the use of insulin glargine is also associated with some adverse events (ADEs). Common ADEs related to insulin glargine include hypoglycemia, weight gain, and injection site reactions (Kişioğlu et al., 2021; Saboo et al., 2024). Furthermore, with the widespread use of this drug in the market, increasing concerns have emerged regarding its real-world safety profile. One study suggested that insulin glargine may be associated with an increased risk of breast cancer (Suissa et al., 2011). A case report from Taiwan described the occurrence of stiff-person syndrome following subcutaneous insulin injection (Lee and Ahn, 2020). Additionally, studies from both the United States and Taiwan have reported insulin-induced amyloidosis (Carll and Antic, 2020; Chen and Lee, 2020). However, most of these studies were systematic reviews, case reports, or RCTs, which are limited by small sample sizes, short follow-up periods, and stringent inclusion and exclusion criteria. Given the extensive global use of insulin glargine since its approval, understanding its real-world safety profile is crucial for assisting clinicians in ensuring the safe use of this medication.

The FDA Adverse Event Reporting System (FAERS) is a public database that collects ADE reports spontaneously submitted by physicians, pharmacists, paramedics, and patients, playing a crucial role in post-marketing drug safety monitoring (Chen S. et al., 2024; Zou et al., 2024). Due to its publicly available large volume of data and real-world data characteristics, an increasing number of researchers have explored the real-world safety of drugs through the FAERS database (Zhao et al., 2024; He et al., 2025). This study employs four disproportionality analysis methods to analyze reports related to insulin glargine in the FAERS database, aiming to provide real-world safety information of insulin glargine for clinicians and regulatory agencies.

## 2 Methods

### 2.1 Data source and process

**FIGURE 1:**
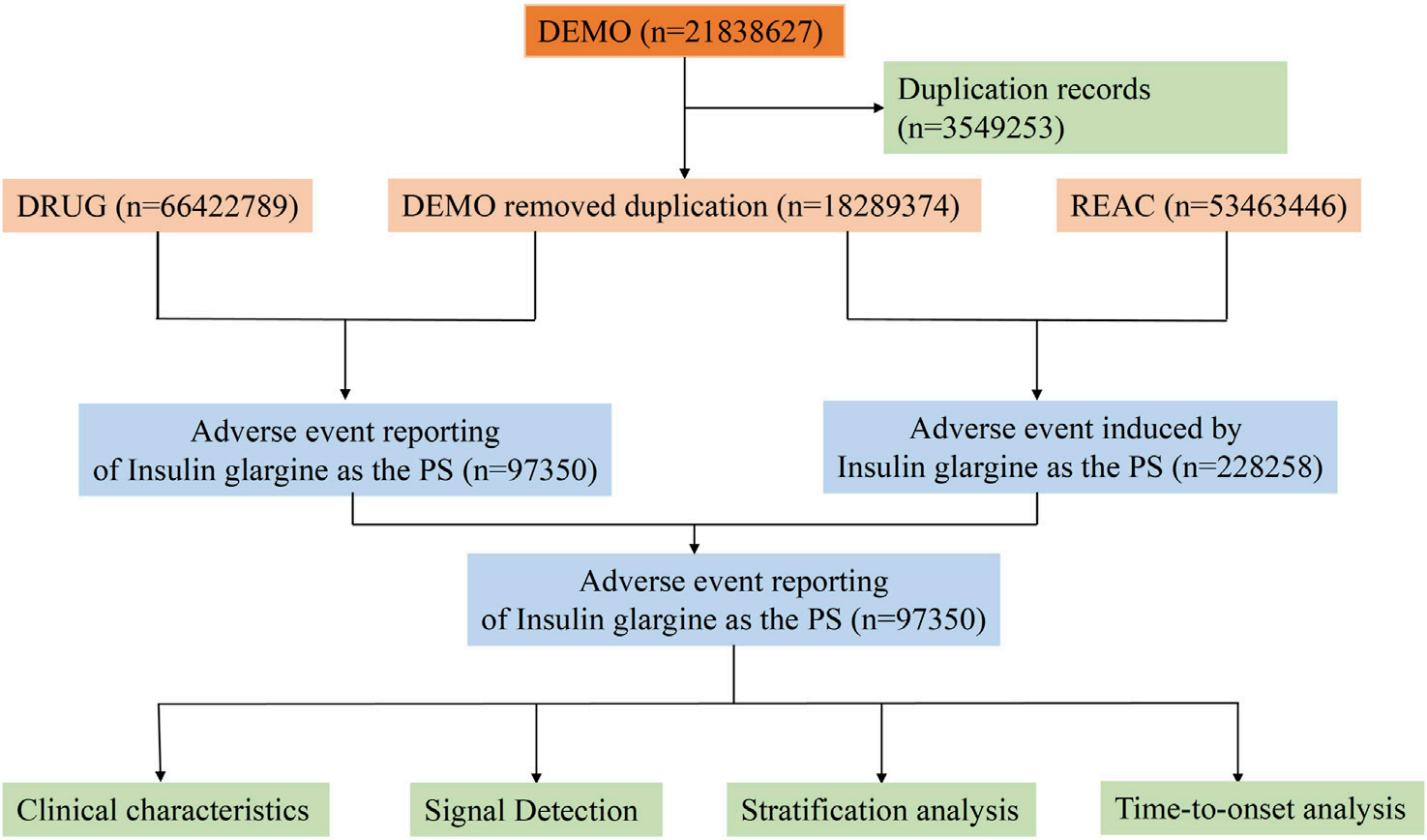
Flowchart of the whole study.

The data for this study was sourced from the FAERS database (https://fis.fda.gov/extensions/FPD-QDE-FAERS/FPD-QDE-FAERS.html). The FAERS database is updated quarterly and contains seven datasets: demographic and administrative information (DEMO), drug information (DRUG), ADEs information (REAC), patient outcome information (OUCT), reporting source information (RPSR), therapy information (THER), and indications for drug administration (INDI). The relationship between drugs and ADE reports is categorized into primary suspected (PS), secondary suspected (SS), concomitant (C), and interaction (I). We collected ADE reports from Q1 2004 to Q3 2024 where “insulin glargine” was listed as the PS. Given that duplicate reports may exist in the FAERS database, we followed the FDA’s deduplication principles (Sakaeda et al., 2013). Specifically, if the case identification (CASEID) values were the same, the report with the largest FDA date (FDA_DT) value was retained. If both CASEID and FDA_DT values were identical, the report with the largest primary identification (PRIMARYID) value was retained. We then standardized the ADEs in the reports using the Medical Dictionary for Regulatory Activities (MedDRA, Version 27.0), primarily mapping the data to the preferred term (PT) and system organ class (SOC) levels. The detailed study process is shown in Figure 1.

### 2.2 Classification criteria for serious adverse events (SADEs)

The severity of ADEs was classified according to Council for International Organizations of Medical Sciences (CIOMS) criteria. Serious adverse events (SADEs) were categorized as follows: DE (Death), LT (Life-Threatening), HO (Hospitalization - Initial or Prolonged), DS (Disability), CA (Congenital Anomaly), RI (Required Intervention to Prevent Permanent Impairment/Damage), and OT (Other Serious - Important Medical Event).

### 2.3 Statistical analysis

#### 2.3.1 Disproportionality analysis

This study employed four disproportionality analysis methods to identify positive signals, including the Reporting Odds Ratio (ROR), Proportional Reporting Ratio (PRR), Bayesian Confidence Propagation Neural Network (MCPNN), and Multi-Item Gamma Poisson Shrinker (MGPS). The ROR and PRR are the most classical disproportionality analysis methods, widely applied in drug safety surveillance due to their broad applicability and ease of implementation. However, these methods are prone to reporting bias and confounding factors, with limited capability in detecting rare ADEs, potentially resulting in false-positive outcomes. The MCPNN and MGPS are more suitable for large-scale data analysis, as they account for multiple variables such as patient age, gender, and concomitant medication use. This improves the accuracy of signal detection and reduces the occurrence of false positives. In this study, an ADE was defined as a positive signal only when it met the criteria of all four disproportionality analysis methods. This stringent criterion significantly improved detection accuracy, reduced false-positive rates, and enhanced the reliability of the results. The fundamental calculation principles of these four methods are presented in Table 1, while their specific computational rules and threshold criteria are detailed in Table 2.

**TABLE 1:** Two-by-two contingency table for disproportionality analyses.

	Target ADEs	Other ADEs	Total
Insulin glargine	a	b	A+ b
Other drugs	c	d	c+d
Total	a+c	b + d	a+b+c+d

Abbreviation: ADEs, adverse events; a, number of reports containing both the target drug and target adverse drug reaction; b, number of reports containing other adverse drug reaction of the target drug; c, number of reports containing the target adverse drug reaction of other drugs; d, number of reports containing other drugs and other adverse drug reactions.

**TABLE 2:** Four major algorithms used for signal detection.

Algorithms	Equation	Criteria
ROR	ROR = ad/b/c	lower limit of 95% CI > 1, N ≥ 3
	95%CI = e^ln(ROR)±1.96(1/a+1/b+1/c+1/d)^0.5^	
PRR	PRR = a (c+d)/c/(a+b)	PRR≥2, χ^2^ ≥ 4, N ≥ 3
	χ^2^ = [(ad-bc)^2](a+b+c+d)/[(a+b) (c+d) (a+c) (b+d)]	
BCPNN	IC = log_2_a (a+b+c+d) (a+c) (a+b)	IC025 > 0
	95%CI = E (IC) ± 2V(IC)^0.5	
MGPS	EBGM = a (a+b+c+d)/(a+c)/(a+b)	EBGM05 > 2
	95%CI = e^ln(EBGM)±1.96(1/a+1/b+1/c+1/d)^0.5^	

Abbreviation: a, number of reports containing both the target drug and target adverse drug reaction; b, number of reports containing other adverse drug reaction of the target drug; c, number of reports containing the target adverse drug reaction of other drugs; d, number of reports containing other drugs and other adverse drug reactions. 95%CI, 95% confidence interval; N, the number of reports; χ2, chi-squared; IC, information component; IC025, the lower limit of 95% CI, of the IC; E (IC), the IC, expectations; V(IC), the variance of IC; EBGM, empirical Bayesian geometric mean; EBGM05, the lower limit of 95% CI, of EBGM.

#### 2.3.2 Time to onset (TTO) and weibull distribution analysis

The TTO of ADEs related to insulin glargine was defined as the time from treatment initiation to the occurrence of the ADEs. The TTO was described using the median and interquartile range (IQR). Additionally, Weibull distribution analysis was conducted to assess the time trend of ADEs. The shape parameter (β) was used to interpret the dynamic changes in failure rates over time. When the β value is <1 and the 95% confidence interval (CI) is also <1, it indicates an early failure trend, where ADEs frequency initially increases but decreases over time. If β = 1 and the 95% CI includes 1, it suggests a constant failure rate, meaning the risk of ADEs remains stable throughout the treatment period. Conversely, when the β value is >1 and the 95% CI excludes 1, it reflects a wear-out failure pattern, indicating that the risk of ADEs increases significantly with prolonged treatment duration.

#### 2.3.3 Sensitivity analysis

To rigorously evaluate the independent safety profile of insulin glargine in real-world settings, this study excluded reports involving the three most frequently co-administered medications: glimepiride, metformin, and sitagliptin. A subsequent disproportionality analysis was performed to minimize potential confounding effects and reduce the impact of drug interactions, thereby enhancing the robustness and reliability of the findings.

## 3 Results

### 3.1 Basic characteristics of insulin glargine ADE reports

This study included 97,350 ADE reports. Among these reports, 51.90% involved female patients, while 38.40% involved male patients. The majority of reports were submitted by individuals aged 65–85 years, accounting for 31.38%. Reports from the United States comprised 85.7% of the total. Regarding ADE outcomes, 13.0% were classified as “Hospitalization - Initial or Prolonged.” Detailed information on other ADE reports is provided in Table 3.

**TABLE 3:** Clinical characteristics of insulin glargine ADE reports from the FAERS database (Q1 2004 – Q3 2024).

Characteristics	Case numbers	Case proportion (%)
Number of events	97,350	100.00%
Gender		
Male	37,409	38.40%
Female	50,567	51.90%
Miss	9,374	9.60%
Age		
<18	930	1.00%
18–65	25,512	26.20%
65–85	30,536	31.38.00%
>85	2,318	2.40%
Miss	38,054	39.10%
Top 5 Reported Countries		
United States	83,453	85.70%
Brazil	1999	2.10%
Egypt	965	1.00%
Japan	913	0.90%
Spain	861	0.90%
Reporter		
Consumer	79,424	81.60%
Nurse	4,092	4.20%
Lawyer	22	0.00%
Physician	4,539	4.70%
Other health professional	2,964	3.00%
Pharmacist	4,634	4.80%
Missing	1,674	1.70%
Reporting year		
2024	7,510	7.71%
2023	7,650	7.86%
2022	8,342	8.57%
2021	8,548	8.78%
2020	8,179	8.40%
2019	9,849	10.12%
2018	12,139	12.47%
2017	5,173	5.31%
2016	4,165	4.28%
2015	6,323	6.50%
2014	6,021	6.18%
2013	3,733	3.83%
2012	1,446	1.49%
2011	1862	1.91%
2010	2,343	2.41%
2009	1,220	1.25%
2008	730	0.75%
2007	792	0.81%
2006	512	0.53%
2005	485	0.50%
2004	328	0.34%
Serious Outcomes		
Death	3,024	3.10%
Disability	1,374	1.40%
Hospitalization - Initial or Prolonged	12,654	13.00%
Life-Threatening	1,039	1.00%
Congenital Anomaly	84	0.1% <
Damage	41	0.1% <
Other Serious	19,694	20.2%
Missing	59,440	61.0%

### 3.2 Disproportionality analysis of ADEs based at SOC level

**FIGURE 2:**
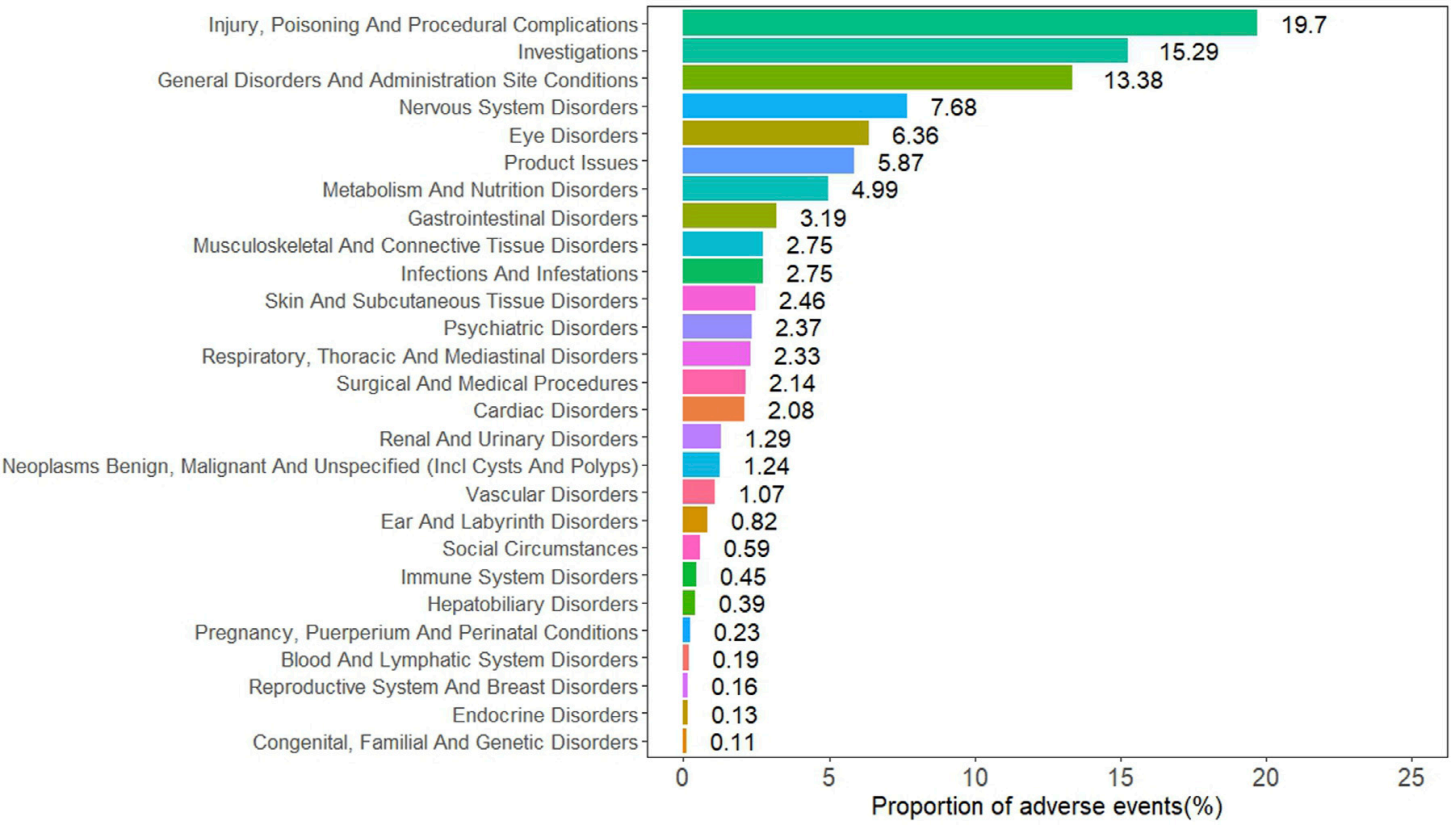
Distribution of ADEs based at SOC level.

At the SOC level, the ADEs are distributed across 24 SOC, with the specific signal values for these ADEs shown in Table 4. The five SOC with the highest number of reports were general disorders and administration site conditions, nervous system disorders, eye disorders, metabolism and nutrition disorders, and gastrointestinal disorders. The distribution of ADEs at the SOC level is shown in Figure 2. Among all the SOC, positive signals were observed for eye disorders and metabolism and nutrition disorders.

**TABLE 4:** Signal strength of insulin glargine ADEs across system organ classes (SOC) in the FAERS database.

SOC	Numbers	ROR (95%CI)	PRR (χ2)	EBGM (EBGM0)	IC (IC025)
Injury, Poisoning And Procedural Complications	44,965	2.37 (2.35–2.4)	2.1 (28,375.07)	2.09 (2.07)	1.06 (1.05)
General Disorders And Administration Site Conditions	30,540	0.72 (0.72–0.73)	0.76 (2760.02)	0.76 (0.75)	−0.39 (−0.41)
Nervous System Disorders	17,523	0.89 (0.87–0.9)	0.9 (226.53)	0.9 (0.89)	−0.16 (−0.18)
Eye Disorders	14,521	3.32 (3.27–3.38)	3.18 (21,798.48)	3.15 (3.1)	1.65 (1.63)
Metabolism And Nutrition Disorders	11,395	2.39 (2.34–2.43)	2.32 (8636.82)	2.3 (2.27)	1.2 (1.18)
Gastrointestinal Disorders	7,274	0.35 (0.34–0.36)	0.37 (8589.14)	0.37 (0.36)	−1.44 (−1.47)
Infections And Infestations	6,277	0.5 (0.49–0.52)	0.52 (2989.31)	0.52 (0.51)	−0.95 (−0.99)
Musculoskeletal And Connective Tissue Disorders	6,271	0.5 (0.49–0.51)	0.51 (3020.8)	0.52 (0.51)	−0.95 (−0.99)
Skin And Subcutaneous Tissue Disorders	5,616	0.44 (0.43–0.45)	0.45 (3912.25)	0.45 (0.44)	−1.14 (−1.18)
Psychiatric Disorders	5,413	0.4 (0.39–0.41)	0.41 (4833.16)	0.41 (0.4)	−1.28 (−1.32)
Respiratory, Thoracic And Mediastinal Disorders	5,327	0.47 (0.46–0.48)	0.48 (3076.93)	0.48 (0.47)	−1.04 (−1.08)
Surgical And Medical Procedures	4,876	1.58 (1.54–1.63)	1.57 (1020.42)	1.57 (1.53)	0.65 (0.61)
Cardiac Disorders	4,759	0.77 (0.75–0.8)	0.78 (308.31)	0.78 (0.76)	−0.36 (−0.4)
Renal And Urinary Disorders	2,938	0.69 (0.66–0.71)	0.69 (409.75)	0.69 (0.67)	−0.53 (−0.58)
Neoplasms Benign, Malignant And Unspecified (Incl Cysts And Polyps)	2,826	0.45 (0.44–0.47)	0.46 (1821.04)	0.46 (0.45)	−1.11 (−1.17)
Vascular Disorders	2,444	0.49 (0.47–0.5)	0.49 (1318.41)	0.49 (0.48)	−1.02 (−1.08)
Ear And Labyrinth Disorders	1882	1.91 (1.83–2)	1.9 (803.08)	1.9 (1.82)	0.92 (0.86)
Immune System Disorders	1,017	0.39 (0.37–0.42)	0.4 (940.09)	0.4 (0.38)	−1.33 (−1.42)
Hepatobiliary Disorders	895	0.42 (0.4–0.45)	0.42 (703.74)	0.43 (0.4)	−1.23 (−1.33)
Pregnancy, Puerperium And Perinatal Conditions	514	0.52 (0.47–0.56)	0.52 (231.4)	0.52 (0.48)	−0.95 (−1.07)
Blood And Lymphatic System Disorders	443	0.11 (0.1–0.12)	0.11 (3153.03)	0.11 (0.1)	−3.15 (−3.28)
Reproductive System And Breast Disorders	355	0.19 (0.17–0.21)	0.19 (1266.09)	0.19 (0.17)	−2.42 (−2.57)
Endocrine Disorders	295	0.5 (0.45–0.56)	0.5 (144.39)	0.5 (0.46)	−0.99 (−1.15)
Congenital, Familial And Genetic Disorders	240	0.34 (0.3–0.39)	0.34 (306.47)	0.34 (0.31)	−1.55 (−1.73)

Abbreviation: ROR, reporting odds ratio; PRR, proportional reporting ratio; EBGM, empirical Bayesian geometric mean; EBGM05, the lower limit of the 95% CI, of EBGM; IC, information component; IC025, the lower limit of the 95% CI, of the IC; CI, confidence interval; ADEs, adverse events.

### 3.3 Disproportionality analysis of ADEs based at PT level

This study identified a total of 130 positive ADEs, with their frequencies and corresponding signal strengths presented in Table 5. The most common positive ADEs include visual impairment, hypoglycemia, injection site pain, hyperglycemia, and cerebrovascular accident. The study confirmed several known ADEs, such as hypoglycemia, injection site pain, injection site hemorrhage, and acquired lipodystrophy. In addition, the study identified some drug-related ADEs not mentioned in the product label, such as pancreatic neoplasm, medullary thyroid cancer, visual impairment, malignant neoplasm of the eye, and bone marrow tumor cell infiltration.

**TABLE 5:** All positive ADEs at the PT Level in the FAERS database.

PT	Case reports	ROR (95%CI)	PRR (χ2)	EBGM (EBGM05)	IC (IC025)
Visual Impairment*	5,850	13.92 (13.55–14.3)	13.59 (64,579.48)	12.89 (12.61)	3.69 (3.65)
Hypoglycaemia	3,718	21.9 (21.17–22.65)	21.56 (66,754.7)	19.81 (19.26)	4.31 (4.26)
Injection Site Pain	3,613	3.4 (3.29–3.52)	3.36 (5944.83)	3.33 (3.24)	1.74 (1.69)
Hyperglycaemia	2,474	19.25 (18.47–20.06)	19.05 (39,132.06)	17.68 (17.08)	4.14 (4.08)
Cerebrovascular Accident	2026	3.09 (2.96–3.23)	3.07 (2800.74)	3.04 (2.93)	1.61 (1.54)
Diabetes Mellitus Inadequate Control	1,596	26.29 (24.96–27.69)	26.11 (34,656.87)	23.57 (22.57)	4.56 (4.48)
Cataract	1,531	7.21 (6.85–7.59)	7.17 (7890.44)	6.98 (6.69)	2.8 (2.73)
Hypoacusis	1,328	8.57 (8.11–9.05)	8.52 (8512.84)	8.26 (7.89)	3.05 (2.96)
Injection Site Haemorrhage	1,271	4.4 (4.16–4.65)	4.38 (3258.03)	4.32 (4.12)	2.11 (2.03)
Memory Impairment	1,135	2.16 (2.04–2.29)	2.15 (697.34)	2.14 (2.04)	1.1 (1.01)
Hyperhidrosis	1,075	2.17 (2.04–2.3)	2.16 (665.95)	2.15 (2.04)	1.1 (1.02)
Injection Site Bruising	1,003	3.53 (3.32–3.76)	3.52 (1785.39)	3.48 (3.31)	1.8 (1.71)
Blindness	986	6.68 (6.27–7.12)	6.66 (4609.6)	6.5 (6.16)	2.7 (2.61)
Injury Associated With Device	839	12.19 (11.37–13.07)	12.15 (8161.71)	11.6 (10.94)	3.54 (3.43)
Cardiac Disorder	812	2.24 (2.09–2.4)	2.24 (552.62)	2.23 (2.1)	1.16 (1.05)
Visual Acuity Reduced	790	5.95 (5.54–6.39)	5.93 (3161.74)	5.81 (5.48)	2.54 (2.43)
Eye Disorder	746	6.21 (5.77–6.68)	6.19 (3163.8)	6.06 (5.7)	2.6 (2.49)
Renal Disorder	643	3.65 (3.37–3.94)	3.64 (1212.25)	3.6 (3.37)	1.85 (1.73)
Diabetic Ketoacidosis	625	7 (6.47–7.59)	6.99 (3114.34)	6.81 (6.37)	2.77 (2.65)
Dementia	448	4.5 (4.1–4.94)	4.49 (1193.06)	4.42 (4.09)	2.15 (2.01)
Glaucoma	391	5.42 (4.9–5.99)	5.41 (1375.6)	5.31 (4.89)	2.41 (2.26)
Eye Haemorrhage	369	7.29 (6.58–8.09)	7.28 (1940.06)	7.09 (6.5)	2.83 (2.67)
Macular Degeneration	360	8.42 (7.58–9.36)	8.41 (2268.55)	8.15 (7.46)	3.03 (2.87)
Injection Site Mass	338	2.49 (2.24–2.77)	2.49 (297.7)	2.47 (2.26)	1.31 (1.15)
Blindness Unilateral	321	6.18 (5.54–6.91)	6.18 (1357.1)	6.04 (5.51)	2.6 (2.43)
Diabetic Retinopathy	296	25.15 (22.31–28.36)	25.12 (6185.58)	22.76 (20.59)	4.51 (4.33)
Ketoacidosis	266	9.61 (8.5–10.86)	9.6 (1968.04)	9.26 (8.35)	3.21 (3.03)
Localised Infection	231	2.51 (2.21–2.86)	2.51 (208.34)	2.5 (2.24)	1.32 (1.13)
Hypoglycaemic Coma	230	22.53 (19.67–25.79)	22.5 (4308.59)	20.6 (18.4)	4.36 (4.17)
Back Disorder	205	3.31 (2.88–3.8)	3.31 (325.37)	3.27 (2.92)	1.71 (1.51)
Hypoglycaemic Unconsciousness	201	27.81 (24.02–32.19)	27.78 (4635.08)	24.92 (22.05)	4.64 (4.43)
Dementia Alzheimer'S Type	194	5.57 (4.83–6.43)	5.57 (710.27)	5.46 (4.85)	2.45 (2.24)
Injection Site Discolouration	184	4.05 (3.5–4.69)	4.05 (414.96)	3.99 (3.54)	2 (1.78)
Retinopathy	183	13.26 (11.43–15.39)	13.25 (1961.02)	12.59 (11.11)	3.65 (3.44)
Diabetic Neuropathy	161	8.45 (7.22–9.89)	8.44 (1019.58)	8.18 (7.17)	3.03 (2.8)
Cold Sweat	159	2.36 (2.02–2.76)	2.36 (123.67)	2.35 (2.06)	1.23 (1)
Hunger	158	3.72 (3.18–4.35)	3.71 (308.55)	3.67 (3.22)	1.88 (1.65)
Injection Site Injury	151	9.04 (7.68–10.63)	9.03 (1037.92)	8.73 (7.62)	3.13 (2.89)
Diabetic Coma	144	13.22 (11.18–15.64)	13.21 (1538.32)	12.56 (10.91)	3.65 (3.4)
Retinal Detachment	139	4.09 (3.46–4.84)	4.09 (319.22)	4.04 (3.51)	2.01 (1.77)
Pancreatic Disorder	138	7.99 (6.74–9.46)	7.98 (814.97)	7.75 (6.72)	2.95 (2.71)
Diabetic Foot	132	11.33 (9.51–13.5)	11.33 (1184.85)	10.84 (9.37)	3.44 (3.18)
Injection Site Extravasation	130	2.56 (2.15–3.04)	2.56 (122.18)	2.54 (2.2)	1.35 (1.09)
Frustration Tolerance Decreased	123	4.24 (3.55–5.07)	4.24 (299.29)	4.18 (3.6)	2.06 (1.8)
Diabetic Metabolic Decompensation	120	25.08 (20.77–30.28)	25.07 (2502.65)	22.72 (19.41)	4.51 (4.23)
Hypoglycaemic Seizure	118	38.16 (31.41–46.37)	38.14 (3665.51)	32.9 (27.95)	5.04 (4.76)
Retinal Haemorrhage	113	4.23 (3.51–5.1)	4.23 (273.6)	4.17 (3.57)	2.06 (1.79)
Diabetic Complication	110	11.1 (9.17–13.45)	11.1 (964.78)	10.64 (9.06)	3.41 (3.13)
Injection Site Discomfort	95	2.67 (2.18–3.27)	2.67 (98.08)	2.65 (2.24)	1.41 (1.11)
Insulin Resistance	86	11.7 (9.42–14.53)	11.69 (800.61)	11.18 (9.33)	3.48 (3.17)
Gangrene	84	3.55 (2.86–4.4)	3.55 (151.49)	3.51 (2.93)	1.81 (1.5)
Infarction	71	2.56 (2.02–3.23)	2.56 (66.52)	2.54 (2.09)	1.34 (1)
Retinal Disorder	67	6.85 (5.38–8.74)	6.85 (325.34)	6.69 (5.46)	2.74 (2.39)
Injection Site Scar	59	5.8 (4.48–7.51)	5.8 (228.65)	5.68 (4.58)	2.51 (2.13)
Shock Hypoglycaemic	50	29.46 (21.95–39.54)	29.45 (1219.56)	26.25 (20.52)	4.71 (4.29)
Diabetic Hyperglycaemic Coma	47	43.47 (31.83–59.36)	43.46 (1642.13)	36.76 (28.33)	5.2 (4.75)
Diabetic Nephropathy	47	5.42 (4.06–7.24)	5.42 (165.62)	5.32 (4.18)	2.41 (1.99)
Injection Site Atrophy	43	6.79 (5.01–9.19)	6.78 (206.04)	6.62 (5.14)	2.73 (2.29)
Throat Clearing	43	3.27 (2.42–4.42)	3.27 (66.86)	3.24 (2.52)	1.7 (1.26)
Hypoglycaemia Neonatal	39	6.42 (4.67–8.83)	6.42 (173.8)	6.28 (4.81)	2.65 (2.19)
Lipodystrophy Acquired	38	4.55 (3.3–6.27)	4.55 (103.17)	4.48 (3.42)	2.16 (1.7)
Reading Disorder	36	4.11 (2.95–5.71)	4.11 (83.11)	4.05 (3.08)	2.02 (1.54)
Diabetic Eye Disease	36	25.25 (17.9–35.61)	25.24 (755.92)	22.86 (17.15)	4.51 (4.02)
Hypoglycaemia Unawareness	34	25.29 (17.75–36.04)	25.29 (715.3)	22.9 (17.03)	4.52 (4.01)
Lipohypertrophy	31	13.55 (9.43–19.47)	13.55 (340.44)	12.86 (9.5)	3.68 (3.16)
Ketosis	29	8.82 (6.09–12.78)	8.82 (193.74)	8.53 (6.26)	3.09 (2.56)
Injection Site Hypersensitivity	28	2.9 (2–4.21)	2.9 (34.42)	2.88 (2.11)	1.52 (0.98)
Myopia	27	2.98 (2.04–4.35)	2.98 (35)	2.95 (2.15)	1.56 (1.01)
Hypoglycaemic Encephalopathy	27	19.83 (13.39–29.38)	19.83 (444.77)	18.35 (13.21)	4.2 (3.63)
Dyslexia	26	6.23 (4.22–9.19)	6.23 (111.1)	6.09 (4.4)	2.61 (2.04)
Neuropathic Arthropathy	24	9.72 (6.46–14.63)	9.72 (180.22)	9.37 (6.66)	3.23 (2.64)
Vascular Occlusion	24	2.95 (1.97–4.42)	2.95 (30.59)	2.93 (2.09)	1.55 (0.97)
Pancreatic Neoplasm*	23	5.06 (3.34–7.64)	5.05 (73.22)	4.97 (3.52)	2.31 (1.72)
Fat Tissue Increased	22	4.09 (2.68–6.23)	4.09 (50.43)	4.03 (2.84)	2.01 (1.41)
Injection Site Hypertrophy	22	22.11 (14.27–34.23)	22.1 (404.72)	20.27 (14.06)	4.34 (3.71)
Diabetic Foot Infection	20	3.46 (2.22–5.38)	3.46 (34.4)	3.42 (2.36)	1.77 (1.14)
Hypermetropia	19	5.08 (3.22–8)	5.08 (60.93)	4.99 (3.41)	2.32 (1.67)
Colour Blindness	17	4.84 (2.99–7.83)	4.84 (50.75)	4.76 (3.19)	2.25 (1.56)
Dawn Phenomenon	17	50.59 (29.94–85.48)	50.58 (678.39)	41.71 (26.89)	5.38 (4.64)
Hyperinsulinaemic Hypoglycaemia	17	26.84 (16.25–44.35)	26.84 (379.07)	24.16 (15.87)	4.59 (3.88)
Ketonuria	15	4.8 (2.88–8.01)	4.8 (44.24)	4.72 (3.08)	2.24 (1.51)
Diabetic Retinal Oedema	14	7.32 (4.3–12.46)	7.32 (74.03)	7.12 (4.56)	2.83 (2.08)
Starvation	14	5.41 (3.18–9.18)	5.41 (49.13)	5.31 (3.41)	2.41 (1.65)
Pulmonary Vasculitis	13	8.62 (4.96–15)	8.62 (84.44)	8.35 (5.25)	3.06 (2.28)
Ocular Vascular Disorder	12	3.58 (2.02–6.33)	3.58 (21.97)	3.54 (2.2)	1.82 (1.02)
Cutaneous Amyloidosis	12	44.92 (24.21–83.35)	44.92 (431.74)	37.8 (22.53)	5.24 (4.37)
Hyperglycaemic Unconsciousness	11	23.64 (12.71–43.96)	23.64 (216.44)	21.55 (12.82)	4.43 (3.56)
Injection Site Laceration	11	4.49 (2.47–8.15)	4.49 (29.24)	4.42 (2.68)	2.14 (1.3)
Diabetic Blindness	10	32.24 (16.64–62.46)	32.23 (265.74)	28.42 (16.34)	4.83 (3.9)
Stomach Mass	10	3.42 (1.83–6.39)	3.42 (16.9)	3.39 (2.01)	1.76 (0.88)
Retinopathy Proliferative	10	13.73 (7.26–25.99)	13.73 (111.46)	13.02 (7.64)	3.7 (2.81)
Brain Stem Stroke	9	4.77 (2.46–9.23)	4.77 (26.27)	4.69 (2.7)	2.23 (1.31)
Acetonaemia	8	9.06 (4.47–18.36)	9.06 (55.19)	8.75 (4.85)	3.13 (2.15)
Retinal Vascular Disorder	8	3.74 (1.86–7.53)	3.74 (15.83)	3.7 (2.06)	1.89 (0.92)
Insulin Autoimmune Syndrome	8	7.74 (3.82–15.65)	7.74 (45.41)	7.52 (4.17)	2.91 (1.93)
Diabetic Ketosis	8	7.49 (3.7–15.14)	7.49 (43.56)	7.28 (4.04)	2.86 (1.89)
Diabetic Gastroparesis	8	5.4 (2.68–10.88)	5.4 (28.01)	5.3 (2.95)	2.41 (1.43)
Pancreas Infection	7	5.16 (2.44–10.91)	5.16 (22.95)	5.07 (2.71)	2.34 (1.31)
Diabetic Ketoacidotic Hyperglycaemic Coma	7	9.85 (4.62–20.98)	9.85 (53.37)	9.49 (5.04)	3.25 (2.2)
Cataract Diabetic	7	35.32 (15.95–78.23)	35.32 (202.59)	30.78 (15.83)	4.94 (3.85)
Drug Effect Faster Than Expected	7	6.6 (3.12–14)	6.6 (32.37)	6.45 (3.44)	2.69 (1.65)
Lens Disorder	6	5.36 (2.38–12.03)	5.36 (20.78)	5.26 (2.67)	2.39 (1.29)
Malignant Neoplasm Of Eye*	6	5.83 (2.59–13.1)	5.83 (23.4)	5.71 (2.9)	2.51 (1.41)
Kidney Malformation	6	4.43 (1.98–9.95)	4.43 (15.66)	4.37 (2.22)	2.13 (1.03)
Decreased Insulin Requirement	6	27.85 (11.94–64.95)	27.85 (138.68)	24.97 (12.3)	4.64 (3.49)
Diabetic Glaucoma	6	58.02 (23.72–141.95)	58.02 (268.98)	46.62 (22.05)	5.54 (4.34)
Medullary Thyroid Cancer*	5	4.62 (1.91–11.2)	4.62 (13.92)	4.55 (2.17)	2.19 (0.99)
Benign Pancreatic Neoplasm	5	7.08 (2.91–17.23)	7.08 (25.31)	6.9 (3.28)	2.79 (1.59)
Acanthosis Nigricans	5	7.08 (2.91–17.23)	7.08 (25.31)	6.9 (3.28)	2.79 (1.59)
Postprandial Hypoglycaemia	5	8.6 (3.52–20.99)	8.6 (32.36)	8.32 (3.94)	3.06 (1.85)
Increased Insulin Requirement	5	5.77 (2.38–14.02)	5.77 (19.25)	5.66 (2.69)	2.5 (1.31)
Diabetic Wound	5	19.02 (7.64–47.34)	19.02 (78.91)	17.66 (8.23)	4.14 (2.91)
Sodium Retention	5	6.27 (2.58–15.25)	6.27 (21.58)	6.13 (2.92)	2.62 (1.42)
Eye Degenerative Disorder	4	6.45 (2.39–17.41)	6.45 (17.91)	6.3 (2.74)	2.66 (1.34)
Insulinoma	4	5.99 (2.22–16.16)	5.99 (16.21)	5.86 (2.56)	2.55 (1.24)
Cerebrovascular Stenosis	4	6.83 (2.53–18.45)	6.83 (19.32)	6.66 (2.9)	2.74 (1.42)
Diabetic Foetopathy	4	15.47 (5.62–42.57)	15.47 (50.76)	14.57 (6.25)	3.86 (2.53)
Erythema Induratum	4	8 (2.95–21.68)	8 (23.69)	7.77 (3.37)	2.96 (1.64)
Diabetic Hepatopathy	4	92.84 (29.12–296.01)	92.83 (259.56)	66.6 (25.24)	6.06 (4.57)
Kussmaul Respiration	4	4.79 (1.78–12.88)	4.79 (11.73)	4.71 (2.06)	2.24 (0.93)
Somogyi Phenomenon	3	46.42 (13.44–160.34)	46.42 (111.1)	38.85 (13.77)	5.28 (3.69)
Splenic Neoplasm Malignancy Unspecified	3	5.9 (1.88–18.56)	5.9 (11.91)	5.78 (2.22)	2.53 (1.06)
Bone Marrow Tumour Cell Infiltration*	3	6.05 (1.92–19.05)	6.05 (12.34)	5.93 (2.27)	2.57 (1.1)
Fumbling	3	6.22 (1.97–19.57)	6.22 (12.79)	6.08 (2.33)	2.6 (1.14)
Injection Site Fibrosis	3	6.51 (2.07–20.5)	6.51 (13.6)	6.36 (2.43)	2.67 (1.2)
Congenital Bladder Anomaly	3	5.36 (1.71–16.82)	5.36 (10.39)	5.26 (2.02)	2.39 (0.93)
Abdominal Fat Apron	3	8.6 (2.72–27.21)	8.6 (19.42)	8.32 (3.17)	3.06 (1.58)
Non-Proliferative Retinopathy	3	87.03 (23.09–328.07)	87.03 (185.55)	63.57 (20.94)	5.99 (4.32)
Vessel Puncture Site Injury	3	29.01 (8.74–96.34)	29.01 (72.12)	25.9 (9.49)	4.69 (3.15)
Abnormal Labour	3	5.8 (1.85–18.25)	5.8 (11.63)	5.68 (2.18)	2.51 (1.04)

Abbreviation:ROR, reporting odds ratio; PRR, proportional reporting ratio; EBGM, empirical Bayesian geometric mean; EBGM05, the lower limit of the 95% CI, of EBGM; IC, information component; IC025, the lower limit of the 95% CI, of the IC; CI, confidence interval; PT, preferred term; * The asterisks indicate noteworthy and unexpected adverse events that are not listed in the drug’s label.

### 3.4 ADEs characteristics across different genders and age groups

The top 50 ADEs associated with insulin glargine in men and women were analyzed at the PT level, with the detailed distribution presented in Supplementary Tables S1, S2. In men, the most frequently reported ADEs were visual impairment, hypoglycemia, and cataract. In women, the most frequently reported ADEs were visual impairment, injection site pain, and hypoglycemia. Additionally, we described the most common ADEs across different age groups. Supplementary Tables S3–S5 present the insulin glargine-related ADEs in each age group. In individuals under 18 years old, hypoglycemia was the most frequently reported PT, while in those aged 18–64 years and ≥65 years, visual impairment was the most commonly reported PT.

### 3.5 ADEs characteristics across different countries

This study further analyzed the top 20 most frequently reported ADEs in the three countries with the highest number of reports, namely, the United States, Brazil, and Egypt. In the United States, the most frequently reported ADEs included visual impairment, injection site pain, hypoglycemia, cerebrovascular accident, cataract, etc. In Brazil, the most commonly reported ADEs were hyperglycemia, hypoglycemia, visual impairment, cerebrovascular accident, injection site pain, etc. In Egypt, the most frequently reported ADEs included death, hyperglycemia, disease progression, cerebrovascular accident, liver disorders, etc. The top 20 most frequently reported ADEs in these three countries are presented in Supplementary Table S6.

### 3.6 ADEs characteristics at different drug dosages

In this study, we analyzed the top 20 most frequently reported ADEs associated with insulin glargine at different doses (10 IU, 20 IU, 30 IU, and 40 IU). The specific distribution of the most common ADEs in each dosage group is shown in Supplementary Table S7. Injection site pain, hypoglycemia, and hyperglycemia were the most frequently reported ADEs across all dosage groups. As the dose increased, both the variety and severity of ADEs exhibited an upward trend. In the 30 IU and 40 IU dosage groups, more severe ADEs, such as myocardial infarction, memory impairment, and cerebrovascular accident, were reported.

### 3.7 Analysis of TTO and Weibull distribution

**FIGURE 3:**
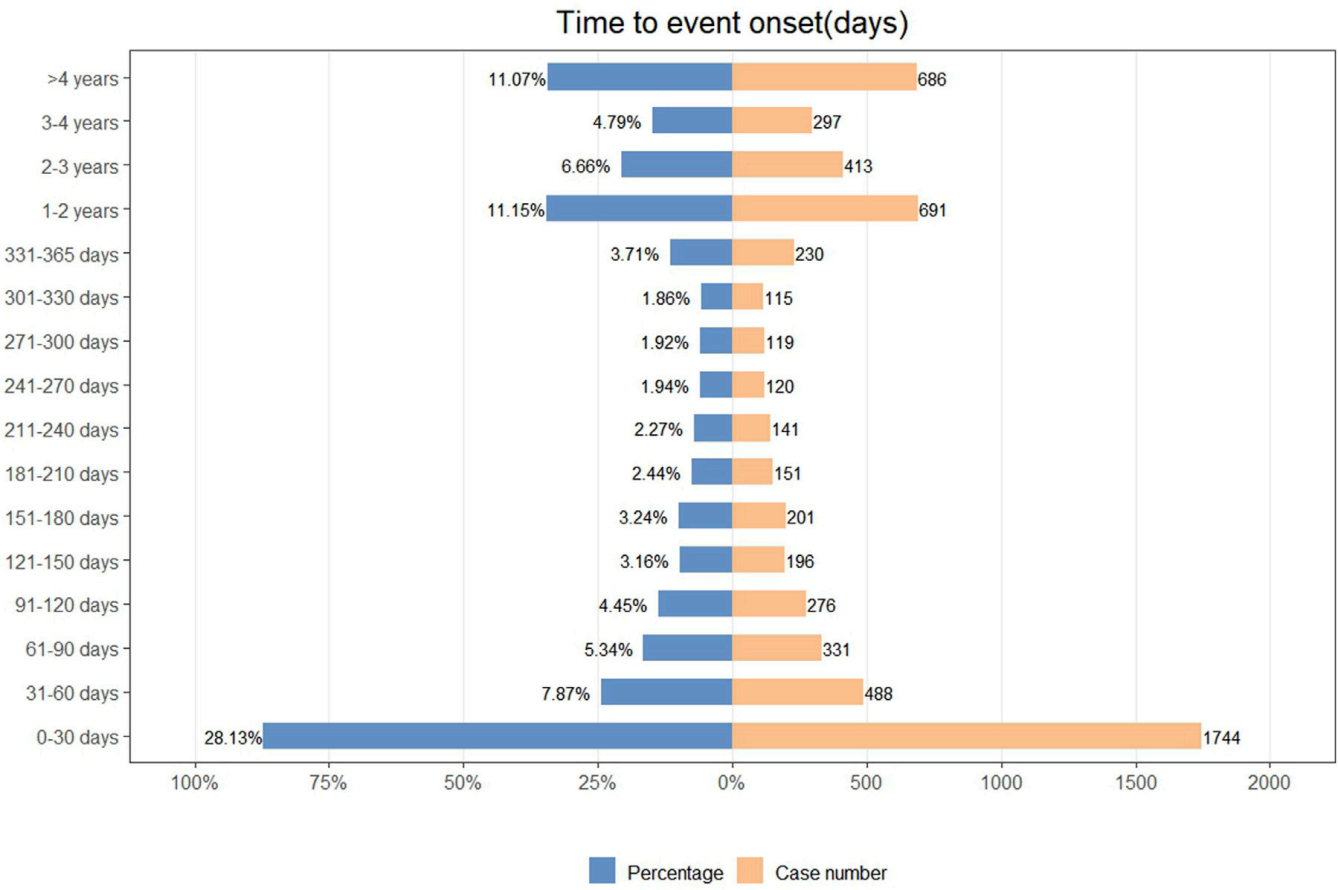
Time to onset of insulin glargine associated ADEs.

We analyzed the TTO of 6199 ADE reports, with the detailed distribution presented in Figure 3. A total of 28.13% of ADEs occurred within the first month, followed by a gradual decline in numbers. Additionally, we employed the Weibull distribution to predict the temporal pattern of ADEs, and the model exhibited an early failure pattern. Further details regarding the parameters are provided in Table 6.

**TABLE 6:** Time to onset of insulin glargine associated ADEs and Weibull distribution analysis.

Drug	TTO (days)		Weibull distribution		
Insulin glargine	Case reports	Median(d) (IQR)	Scale parameter: α(95%CI)	Shape parameter: β(95%CI)	Type
	6,199	158 (24–644)	309.48 (294.31–324.65)	0.54 (0.53–0.55)	Early failure

Abbreviation: TTO, time to onset; CI, confidence interval; IQR, interquartile range.

### 3.8 Sensitivity analysis

Insulin glargine is commonly used in combination with glimepiride, metformin, and sitagliptin. This study further excluded reports involving these concomitant medications and conducted a disproportionality analysis again. A total of 91,698 reports were included, with 209,705 ADEs. The ADEs that continued to show positive signals included hypoglycemia, injection site pain, injection site hemorrhage, acquired lipodystrophy, pancreatic neoplasm, medullary thyroid cancer, visual impairment, malignant neoplasm of the eye, and bone marrow tumor cell infiltration. These findings were largely consistent with the results of the previous overall analysis, with specific signal strengths and frequencies presented in Supplementary Table S8.

## 4 Discussion

This study analyzed the real-world safety of insulin glargine using the FAERS database. The study confirmed several known ADEs, such as hypoglycemia, injection site reactions, and lipodystrophy. Additionally, the study identified some unexpected ADEs, including pancreatic neoplasm, medullary thyroid cancer, malignant neoplasm of the eye, and bone marrow tumor cell infiltration. These findings provide new safety insights for healthcare professionals regarding insulin glargine in real-world settings.

Hypoglycemia typically presents with symptoms such as dizziness, sweating, nausea, and hunger (Tripyla et al., 2023). Several clinical studies and reviews have indicated that hypoglycemia is a common ADE associated with the use of insulin glargine (Sebastian et al., 2023; Chen L. et al., 2024; Hong et al., 2024; Home, 2025). This finding is consistent with the results of the present study. Mild hypoglycemia may only cause symptoms like dizziness, palpitations, and sweating (Tripyla et al., 2023). However, the dangers of hypoglycemia should not be underestimated, as severe hypoglycemia can directly lead to coma, cardiac arrest, or seizures, all of which are potentially fatal (Husain et al., 2023). Furthermore, recurrent hypoglycemia over time may lead to psychological issues such as anxiety and depression, and even increase the risk of cardiovascular ADEs (Li et al., 2014; Hinnen and Kruger, 2019). Fortunately, hypoglycemia, as a common ADE associated with insulin glargine use, has attracted widespread attention from clinicians, and severe ADEs due to hypoglycemia are rare.

Injection site reactions are common ADEs associated with insulin glargine. The injection site reactions that showed positive signals in this study included injection site pain, injection site hemorrhage, injection site bruising, and injection site mass. A multicenter clinical trial demonstrated that injection site reactions are common ADEs with insulin glargine use (Rosenstock et al., 2015). Another RCT study also confirmed that injection site reactions are frequent ADEs (Yan et al., 2022). The findings of this study are consistent with these researches. Fortunately, injection site reactions are typically mild and do not affect patient adherence to treatment. However, it is worth noting that this study also found that local infections showed positive signals, and clinicians should advise patients to ensure proper disinfection before injection to prevent infection.

In addition, this study also identified several unexpected ADEs, including pancreatic neoplasm, medullary thyroid cancer, visual impairment, malignant neoplasm of eye, and bone marrow tumour cell infiltration. Pancreatic neoplasm, particularly pancreatic cancer, can affect both the endocrine and exocrine functions of the pancreas, leading to symptoms such as indigestion, jaundice, and hyperglycemia (Hussain et al., 2022). The progression and metastasis of pancreatic cancer can lead to complications such as ascites, thrombosis, and respiratory distress, and it is often diagnosed at an advanced stage, with an overall 5-year survival rate of approximately 10% (Jiang et al., 2023). A cohort study investigating cancer risks in insulin users across five countries found that, except among Norwegians, pancreatic cancer was reported as the most common cancer (But et al., 2017). A systematic review and meta-analysis also indicated that insulin use is associated with an increased risk of pancreatic cancer (Karlstad et al., 2013). Another systematic review and meta-analysis of observational studies found that new use of insulin glargine is associated with an increased risk of pancreatic cancer (Colmers et al., 2012). These findings are consistent with the results of this study and support the notion that insulin use may be linked to pancreatic cancer. Given the significant impact of pancreatic cancer on patient health, we recommend that pancreatic tumor monitoring should be closely observed during insulin therapy for diabetes to ensure timely detection and management.

Visual impairment is another unexpected ADE. Based on multiple FAERS studies and clinical practice experience (Xiang et al., 2023; Zhang et al., 2024), we believe that the use of insulin glargine in diabetic patients complicates the differentiation between whether visual impairment is an ADE induced by insulin glargine. This is because visual impairment itself may be related to the clinical manifestations of diabetes, such as cataracts or glaucoma, which can lead to visual impairment or even blindness (Grauslund, 2022). However, considering the significant impact of visual impairment on patients’ daily life, work, and education, it can also affect medication adherence (Paudel et al., 2022). Our study recommends that, when using insulin glargine to treat diabetes, close monitoring of patients’ clinical manifestations is essential to prevent further deterioration of vision.

Moreover, medullary thyroid cancer (MTC) is another unexpected ADE that warrants attention. MTC presents clinically with symptoms such as neck masses, hypercalcemia, dysphagia, and hoarseness (Khan et al., 2022). The relationship between insulin glargine and thyroid cancer remains unclear. A retrospective study indicated no significant association between insulin glargine and pancreatic cancer (Abi Zeid Daou et al., 2025). However, animal studies have shown that insulin glargine can promote thyroid cell proliferation through mitogenic signaling pathways (Sheng et al., 2019). Furthermore, cell experiments suggest that insulin glargine may facilitate thyroid cell proliferation and migration through the insulin-like growth factor-1 receptor and the downstream Akt-signaling pathway (Zhang et al., 2019). Our study found a statistical association between insulin glargine and medullary thyroid cancer, though further prospective studies are needed to validate these findings.

Subgroup analysis suggests that in male patients, attention should be given to the occurrence of cataracts, while in female patients, the occurrence of injection site pain should be closely monitored and promptly managed, which may be related to women’s increased sensitivity to pain (Bulls et al., 2015). In patients under 18 years of age, the occurrence of hypoglycemia should be a primary concern. In patients aged 18 and above, visual impairment should be closely monitored, which is more likely associated with long-term diabetic complications rather than the use of insulin glargine.

After analyzing the top 20 ADEs in the United States, Brazil, and Egypt, we found that hypoglycemia and injection site reactions occurred in all three countries, suggesting that these ADEs are not significantly influenced by genetic variation, environmental factors, or healthcare conditions. Additionally, vision-related disorders (e.g., visual impairment, cataract) were more prevalent in the United States and Brazil, while severe metabolic events (e.g., diabetic ketoacidosis, death) were more frequently reported in Egypt. These differences may be influenced by genetic factors, healthcare infrastructure, and public awareness of diabetes management. The differences in ADEs among different countries warrant further investigation.

This study reveals the distribution of ADEs associated with insulin glargine at different doses. Compared to lower doses (e.g., 10 IU), higher doses (e.g., 40 IU) showed significantly different numbers and types of reported ADEs. Common ADEs at lower doses included milder metabolic reactions such as hypoglycemia and hyperglycemia, while with increasing doses, a greater number of metabolic and neurological ADEs were reported, such as myocardial infarction, memory impairment, and cerebrovascular accidents. However, these may not be directly related to the drug dosage itself, but rather due to the fact that patients with higher dosages often have more severe diabetes and may also have comorbid conditions (Russell and Cooper, 2015), making them more likely to experience severe ADEs. Future prospective studies should further explore the ADEs and clinical impacts of insulin glargine at different doses to better guide individualized treatment.

The TTO analysis indicates that the majority of insulin glargine-related ADEs occur within the first month, with a gradual reduction in ADEs thereafter. The Weibull distribution aligns with the early failure model, suggesting that the occurrence of ADEs decreases over time, consistent with the TTO analysis. This study emphasizes the importance of heightened vigilance in monitoring insulin glargine-related ADEs during the first month of use, with continuous monitoring throughout the treatment period to ensure timely management of ADEs and guarantee patient medication safety.

Sensitivity analysis showed that after excluding reports of insulin glargine in combination with glimepiride, metformin, and sitagliptin, a disproportionality analysis was re-conducted. ADEs such as hypoglycemia, injection site pain, injection site hemorrhage, acquired lipodystrophy, pancreatic neoplasm, medullary thyroid cancer, visual impairment, malignant neoplasm of the eye, and bone marrow tumor cell infiltration still exhibited positive signals, reinforcing the robustness of the study’s findings.

This study has several limitations. First, ADE reports primarily come from spontaneous reports by physicians, pharmacists, paramedics, and patients, which may introduce reporting bias (Zhao et al., 2024). Furthermore, as spontaneous reports are prone to underreporting, selective reporting, and misreporting, these reports often lack critical information, such as detailed treatment regimens, drug dosages, duration of use, and patients’ comorbidities, which may affect the accuracy of the results. Therefore, the findings of this study should be interpreted with caution. Additionally, 85.7% of the reports in this study came from the United States, which may affect the external validity of the results. Future research should consider including reports from other countries to conduct broader analyses. Finally, disproportionality analysis methods only reveal statistical associations between drugs and ADEs, but cannot establish causality (Sakaeda et al., 2013). Future large-scale prospective studies are needed to validate the findings of this study.

## 5 Conclusion

This study analyzed ADE reports related to insulin glargine in the FAERS database using four disproportionate analysis methods. The study confirmed several known ADEs, such as hypoglycemia and injection site reactions. The study also identified some unexpected ADEs, such as pancreatic neoplasm and medullary thyroid cancer. These findings provide new insights into the real-world safety of insulin glargine for clinicians and regulatory agencies. Future prospective studies are needed to validate the findings of this study.

## Data Availability

The datasets presented in this study can be found in online repositories. The names of the repository/repositories and accession number(s) can be found in the article/Supplementary Material.
