# Supplementary material for: Adverse drug events associated with insulin glargine: a real-world pharmacovigilance study based on the FAERS database
Source: Front Pharmacol. 2025 Apr 28;16:1563238. doi: 10.3389/fphar.2025.1563238 (PMC12066629; doi:10.3389/fphar.2025.1563238)
Supplement: Supplementary file 1 [file DataSheet1.docx]

Supplementary Material

Supplementary Table 1:

Top 50 most frequent ADEs for insulin glargine at the PT level in males from FAERS data

| PT | Numbers | ROR(95%CI) | PRR(χ^2^) | EBGM  (EBGM05) | IC(IC025) |
| --- | --- | --- | --- | --- | --- |
| Visual Impairment | 1777 | 12.83(12.23-13.47) | 12.59(17907.36) | 11.93(11.46) | 3.58(3.51) |
| Hypoglycaemia | 1646 | 20.63(19.6-21.71) | 20.25(27490.31) | 18.55(17.77) | 4.21(4.14) |
| Hypoacusis | 599 | 8.32(7.67-9.03) | 8.27(3686.54) | 7.99(7.47) | 3(2.88) |
| Hyperhidrosis | 455 | 2.23(2.03-2.44) | 2.22(303.08) | 2.21(2.04) | 1.14(1.01) |
| Cataract | 410 | 6.07(5.5-6.69) | 6.04(1677.51) | 5.9(5.43) | 2.56(2.42) |
| Cardiac Disorder | 395 | 2.23(2.02-2.47) | 2.23(265.33) | 2.22(2.04) | 1.15(1) |
| Blindness | 391 | 6.86(6.2-7.59) | 6.83(1886.58) | 6.65(6.11) | 2.73(2.58) |
| Renal Disorder | 298 | 3.72(3.31-4.17) | 3.71(579.72) | 3.66(3.33) | 1.87(1.7) |
| Injection Site Bruising | 261 | 4.4(3.89-4.98) | 4.39(670.25) | 4.32(3.9) | 2.11(1.93) |
| Visual Acuity Reduced | 250 | 4.98(4.39-5.64) | 4.96(773.41) | 4.87(4.39) | 2.28(2.1) |
| Eye Disorder | 225 | 5.98(5.24-6.83) | 5.97(904.81) | 5.83(5.22) | 2.54(2.35) |
| Injection Site Mass | 152 | 3.3(2.81-3.87) | 3.29(239) | 3.26(2.85) | 1.7(1.47) |
| Glaucoma | 132 | 5.55(4.67-6.6) | 5.54(478.68) | 5.42(4.69) | 2.44(2.19) |
| Localised Infection | 121 | 2.99(2.5-3.58) | 2.99(157.71) | 2.96(2.55) | 1.56(1.3) |
| Eye Haemorrhage | 110 | 6.28(5.19-7.59) | 6.27(473.11) | 6.12(5.22) | 2.61(2.33) |
| Blindness Unilateral | 108 | 5.26(4.34-6.36) | 5.25(362.56) | 5.15(4.39) | 2.36(2.08) |
| Ketoacidosis | 97 | 7.8(6.36-9.55) | 7.79(553.37) | 7.54(6.37) | 2.92(2.62) |
| Macular Degeneration | 90 | 8.24(6.67-10.17) | 8.23(550.27) | 7.96(6.67) | 2.99(2.68) |
| Cold Sweat | 85 | 2.98(2.4-3.69) | 2.98(110.06) | 2.95(2.47) | 1.56(1.25) |
| Injection Site Discolouration | 60 | 6.66(5.15-8.61) | 6.65(279.41) | 6.48(5.23) | 2.7(2.32) |
| Hypoglycaemic Seizure | 58 | 38.12(28.81-50.43) | 38.09(1771.66) | 32.37(25.61) | 5.02(4.61) |
| Retinal Detachment | 58 | 3.7(2.86-4.8) | 3.7(112.35) | 3.65(2.94) | 1.87(1.49) |
| Diabetic Neuropathy | 57 | 6.33(4.86-8.23) | 6.32(247.88) | 6.17(4.95) | 2.62(2.24) |
| Gangrene | 56 | 3.77(2.89-4.91) | 3.77(111.86) | 3.72(2.98) | 1.89(1.51) |
| Diabetic Metabolic Decompensation | 44 | 17.15(12.61-23.33) | 17.14(618.16) | 15.92(12.31) | 3.99(3.55) |
| Retinal Haemorrhage | 40 | 3.31(2.43-4.53) | 3.31(63.61) | 3.28(2.52) | 1.71(1.26) |
| Injection Site Induration | 40 | 3.03(2.21-4.13) | 3.02(53.44) | 3(2.31) | 1.58(1.13) |
| Injection Site Atrophy | 28 | 26.83(18.1-39.77) | 26.82(616.87) | 23.88(17.18) | 4.58(4.01) |
| Lipohypertrophy | 21 | 23.58(15.02-37.03) | 23.58(407.98) | 21.29(14.59) | 4.41(3.77) |
| Skin Odour Abnormal | 20 | 3.29(2.12-5.12) | 3.29(31.46) | 3.26(2.25) | 1.7(1.07) |
| Lipodystrophy Acquired | 19 | 4.58(2.91-7.21) | 4.58(51.97) | 4.5(3.08) | 2.17(1.52) |
| Hypoglycaemia Unawareness | 18 | 25.93(15.89-42.32) | 25.92(383.71) | 23.17(15.38) | 4.53(3.84) |
| Injection Site Hypertrophy | 17 | 62.3(36.24-107.08) | 62.29(789.62) | 48.21(30.64) | 5.59(4.83) |
| Pancreatic Neoplasm | 13 | 6.05(3.48-10.5) | 6.05(53.22) | 5.9(3.72) | 2.56(1.78) |
| Diabetic Eye Disease | 13 | 21.9(12.37-38.78) | 21.89(234.62) | 19.91(12.34) | 4.32(3.51) |
| Ketosis | 12 | 7.31(4.11-12.99) | 7.31(63.11) | 7.09(4.38) | 2.83(2.01) |
| Neuropathic Arthropathy | 12 | 7.98(4.48-14.21) | 7.98(70.58) | 7.72(4.77) | 2.95(2.13) |
| Hypoglycaemic Encephalopathy | 12 | 18.03(10-32.52) | 18.03(177.68) | 16.68(10.18) | 4.06(3.23) |
| Colour Blindness | 11 | 6.05(3.32-11.01) | 6.05(45.02) | 5.9(3.57) | 2.56(1.72) |
| Pancreatic Failure | 9 | 4.75(2.45-9.19) | 4.75(26.02) | 4.66(2.68) | 2.22(1.3) |
| Injection Site Laceration | 8 | 9.33(4.6-18.95) | 9.33(56.98) | 8.98(4.96) | 3.17(2.18) |
| Injection Site Hypersensitivity | 7 | 3.84(1.82-8.1) | 3.84(14.42) | 3.79(2.03) | 1.92(0.89) |
| Ketonuria | 7 | 4.25(2.01-8.98) | 4.25(17.05) | 4.18(2.24) | 2.07(1.03) |
| Injection Site Ulcer | 6 | 4.37(1.95-9.8) | 4.37(15.25) | 4.3(2.18) | 2.1(1) |
| Pancreas Infection | 6 | 7.16(3.17-16.16) | 7.16(30.75) | 6.96(3.52) | 2.8(1.69) |
| Ocular Vascular Disorder | 6 | 5.33(2.37-11.99) | 5.33(20.59) | 5.22(2.65) | 2.39(1.28) |
| Brain Stem Stroke | 6 | 6.2(2.75-13.97) | 6.2(25.43) | 6.05(3.07) | 2.6(1.49) |
| Diabetic Retinal Oedema | 6 | 7.29(3.23-16.44) | 7.29(31.44) | 7.07(3.58) | 2.82(1.71) |
| Insulin Autoimmune Syndrome | 6 | 8.76(3.87-19.83) | 8.76(39.6) | 8.45(4.27) | 3.08(1.96) |
| Diabetic Blindness | 5 | 24.28(9.62-61.31) | 24.28(100) | 21.86(10.07) | 4.45(3.2) |

Abbreviation: ROR, reporting odds ratio; PRR, proportional reporting ratio; EBGM, empirical Bayesian geometric mean; EBGM05, the lower limit of the 95% CI of EBGM; IC, information component; IC025, the lower limit of the 95% CI of the IC; CI, confidence interval; PT,preferred term; ADEs, adverse events.

Supplementary Table 2:

Top 50 most frequent ADEs for insulin glargine at the PT level in females from FAERS data

| **PT** | **Case numbers** | **ROR(95%CI)** | **PRR(χ^2^)** | **EBGM**  **(EBGM05)** | | **IC(IC025)** |
| --- | --- | --- | --- | --- | --- | --- |
| Visual Impairment | 3760 | 14.71(14.23-15.21) | 14.3(43994.85) | 13.55(13.18) | 3.76(3.71) | |
| Injection Site Pain | 2123 | 3.03(2.9-3.16) | 2.99(2799.92) | 2.97(2.86) | 1.57(1.51) | |
| Hypoglycaemia | 1804 | 22.31(21.25-23.42) | 22(33149.64) | 20.24(19.43) | 4.34(4.27) | |
| Cataract | 1074 | 8.15(7.67-8.67) | 8.09(6464.51) | 7.86(7.47) | 2.97(2.88) | |
| Cerebrovascular Accident | 1013 | 3.19(3-3.4) | 3.17(1493.31) | 3.15(2.99) | 1.65(1.56) | |
| Injection Site Haemorrhage | 737 | 3.98(3.7-4.28) | 3.97(1610.14) | 3.92(3.68) | 1.97(1.86) | |
| Memory Impairment | 714 | 2.16(2-2.32) | 2.15(437.32) | 2.14(2.01) | 1.1(0.99) | |
| Injection Site Bruising | 666 | 3.11(2.88-3.36) | 3.1(936.67) | 3.07(2.88) | 1.62(1.51) | |
| Hypoacusis | 640 | 8.16(7.54-8.83) | 8.12(3869.4) | 7.89(7.39) | 2.98(2.86) | |
| Injury Associated With Device | 575 | 12.1(11.13-13.16) | 12.05(5550.94) | 11.52(10.74) | 3.53(3.4) | |
| Blindness | 541 | 6.89(6.33-7.51) | 6.87(2638.39) | 6.7(6.24) | 2.75(2.62) | |
| Visual Acuity Reduced | 532 | 7.17(6.58-7.82) | 7.14(2731.46) | 6.97(6.48) | 2.8(2.67) | |
| Eye Disorder | 498 | 6.72(6.15-7.35) | 6.7(2350.67) | 6.54(6.07) | 2.71(2.58) | |
| Cardiac Disorder | 392 | 2.36(2.14-2.61) | 2.36(303.15) | 2.34(2.15) | 1.23(1.08) | |
| Diabetic Ketoacidosis | 335 | 8.11(7.27-9.04) | 8.09(2014.54) | 7.86(7.17) | 2.97(2.81) | |
| Renal Disorder | 320 | 3.75(3.36-4.19) | 3.74(634.13) | 3.7(3.37) | 1.89(1.73) | |
| Macular Degeneration | 260 | 8.97(7.92-10.15) | 8.95(1770.4) | 8.66(7.81) | 3.12(2.93) | |
| Eye Haemorrhage | 255 | 8.96(7.91-10.16) | 8.95(1736.04) | 8.66(7.8) | 3.11(2.93) | |
| Glaucoma | 250 | 5.77(5.09-6.54) | 5.76(960.93) | 5.65(5.09) | 2.5(2.31) | |
| Dementia | 220 | 4.4(3.85-5.03) | 4.39(566.71) | 4.33(3.88) | 2.12(1.92) | |
| Blindness Unilateral | 195 | 7.11(6.17-8.2) | 7.1(993.33) | 6.93(6.15) | 2.79(2.58) | |
| Diabetic Retinopathy | 194 | 33.35(28.7-38.76) | 33.3(5337.85) | 29.36(25.9) | 4.88(4.66) | |
| Ketoacidosis | 147 | 11.57(9.8-13.65) | 11.55(1352.29) | 11.07(9.64) | 3.47(3.23) | |
| Back Disorder | 117 | 3.11(2.59-3.74) | 3.11(165.48) | 3.08(2.65) | 1.62(1.36) | |
| Injection Site Discolouration | 116 | 3.39(2.82-4.07) | 3.39(192.54) | 3.35(2.88) | 1.75(1.48) | |
| Retinopathy | 111 | 16.06(13.25-19.46) | 16.05(1467.9) | 15.1(12.86) | 3.92(3.64) | |
| Dementia Alzheimer'S Type | 106 | 5.48(4.52-6.64) | 5.47(378.79) | 5.37(4.57) | 2.43(2.14) | |
| Thyroid Disorder | 103 | 2.54(2.09-3.09) | 2.54(95.33) | 2.53(2.15) | 1.34(1.05) | |
| Hypoglycaemic Unconsciousness | 99 | 27.66(22.46-34.06) | 27.64(2279.3) | 24.89(20.91) | 4.64(4.33) | |
| Diabetic Neuropathy | 93 | 9.53(7.75-11.73) | 9.53(682.74) | 9.2(7.74) | 3.2(2.9) | |
| Hypoglycaemic Coma | 93 | 18.93(15.33-23.38) | 18.92(1462.9) | 17.61(14.76) | 4.14(3.83) | |
| Diabetic Coma | 92 | 17.09(13.83-21.12) | 17.08(1299.98) | 16.01(13.41) | 4(3.69) | |
| Hunger | 88 | 3.19(2.59-3.94) | 3.19(130.67) | 3.16(2.65) | 1.66(1.35) | |
| Retinal Detachment | 79 | 5.01(4.01-6.26) | 5.01(248.37) | 4.93(4.09) | 2.3(1.97) | |
| Pancreatic Disorder | 75 | 8.17(6.49-10.28) | 8.16(456.04) | 7.93(6.54) | 2.99(2.65) | |
| Retinal Haemorrhage | 67 | 5.67(4.45-7.23) | 5.67(251.76) | 5.56(4.54) | 2.48(2.12) | |
| Injection Site Injury | 66 | 6.29(4.93-8.04) | 6.29(286.22) | 6.16(5.02) | 2.62(2.26) | |
| Frustration Tolerance Decreased | 65 | 3.71(2.91-4.74) | 3.71(126.82) | 3.67(2.99) | 1.88(1.52) | |
| Diabetic Foot | 61 | 16.65(12.84-21.59) | 16.64(838.71) | 15.63(12.58) | 3.97(3.59) | |
| Diabetic Metabolic Decompensation | 59 | 28.03(21.4-36.71) | 28.02(1376.44) | 25.19(20.1) | 4.65(4.26) | |
| Diabetic Complication | 55 | 11.99(9.14-15.71) | 11.98(527.19) | 11.46(9.13) | 3.52(3.12) | |
| Injection Site Discomfort | 55 | 2.61(2-3.41) | 2.61(54.18) | 2.6(2.08) | 1.38(0.99) | |
| Hypoglycaemic Seizure | 49 | 34.88(25.85-47.07) | 34.87(1407.43) | 30.57(23.79) | 4.93(4.5) | |
| Retinal Disorder | 45 | 8.91(6.62-12) | 8.91(304.76) | 8.63(6.73) | 3.11(2.68) | |
| Insulin Resistance | 43 | 10.76(7.92-14.6) | 10.75(364.08) | 10.33(8) | 3.37(2.92) | |
| Nephropathy | 43 | 2.76(2.05-3.73) | 2.76(47.81) | 2.74(2.13) | 1.46(1.02) | |
| Infarction | 36 | 3.17(2.28-4.4) | 3.17(52.77) | 3.14(2.39) | 1.65(1.17) | |
| Injection Site Scar | 30 | 4.54(3.16-6.52) | 4.54(81.25) | 4.47(3.31) | 2.16(1.64) | |
| Gangrene | 27 | 3.06(2.09-4.47) | 3.06(36.98) | 3.03(2.21) | 1.6(1.05) | |
| Diabetic Hyperglycaemic Coma | 25 | 47.22(30.75-72.51) | 47.21(944.78) | 39.61(27.67) | 5.31(4.7) | |

Abbreviation: ROR, reporting odds ratio; PRR, proportional reporting ratio; EBGM, empirical Bayesian geometric mean; EBGM05, the lower limit of the 95% CI of EBGM; IC, information component; IC025, the lower limit of the 95% CI of the IC; CI, confidence interval; PT,preferred term; ADE, adverse events.

Supplementary Table 3:

ADEs at the PT level for insulin glargine in patients aged under 18 from FAERS data

| **PT** | **Case numbers** | **ROR(95%CI)** | **PRR(χ^2^)** | **EBGM**  **(EBGM05)** | **IC(IC025)** |
| --- | --- | --- | --- | --- | --- |
| Hypoglycaemia | 105 | 43.54(35.61-53.23) | 41.75(3943.17) | 39.43(33.33) | 5.3(5.01) |
| Diabetic Ketoacidosis | 86 | 87.65(69.82-110.03) | 84.66(6338.85) | 75.55(62.46) | 6.24(5.91) |
| Ketoacidosis | 45 | 197.25(141.4-275.15) | 193.71(6743.37) | 151.61(114.76) | 7.24(6.77) |
| Injection Site Pain | 42 | 3.29(2.42-4.47) | 3.25(65.53) | 3.24(2.51) | 1.7(1.25) |
| Diabetes Mellitus Inadequate Control | 37 | 238.78(164.11-347.43) | 235.25(6443.93) | 175.89(128.52) | 7.46(6.93) |
| Loss Of Consciousness | 18 | 3.38(2.13-5.39) | 3.37(29.87) | 3.36(2.27) | 1.75(1.08) |
| Hypoglycaemic Seizure | 17 | 83.53(50.43-138.35) | 82.97(1229.62) | 74.21(48.65) | 6.21(5.49) |
| Dehydration | 16 | 3.67(2.24-6.01) | 3.65(30.71) | 3.64(2.41) | 1.86(1.16) |
| Coma | 14 | 4.34(2.56-7.35) | 4.32(35.55) | 4.3(2.77) | 2.1(1.35) |
| Diabetic Metabolic Decompensation | 7 | 270.27(112.79-647.67) | 269.52(1348.33) | 194.33(93.53) | 7.6(6.43) |
| Stress | 7 | 6.23(2.96-13.12) | 6.21(30.35) | 6.17(3.31) | 2.62(1.6) |
| Coeliac Disease | 7 | 17.37(8.2-36.82) | 17.33(105.08) | 16.93(9.03) | 4.08(3.05) |
| Acidosis | 6 | 7.4(3.31-16.56) | 7.39(32.79) | 7.32(3.73) | 2.87(1.77) |
| Polydipsia | 6 | 20.53(9.11-46.28) | 20.48(108.02) | 19.92(10.09) | 4.32(3.21) |
| Diabetic Coma | 6 | 106.88(45.21-252.67) | 106.62(544.1) | 92.54(45.05) | 6.53(5.36) |
| Diabetic Hyperglycaemic Coma | 5 | 192.9(71.56-519.97) | 192.51(745.52) | 150.88(65.81) | 7.24(5.92) |
| Trismus | 5 | 11.23(4.64-27.21) | 11.21(45.79) | 11.05(5.27) | 3.47(2.28) |
| Polyuria | 4 | 8.59(3.2-23.06) | 8.58(26.47) | 8.49(3.72) | 3.09(1.78) |
| Hepatic Steatosis | 4 | 6.72(2.51-18.01) | 6.71(19.26) | 6.66(2.92) | 2.73(1.44) |
| Acetonaemia | 4 | 213.59(69.59-655.5) | 213.25(646.18) | 163.31(63.9) | 7.35(5.89) |
| Ketonuria | 4 | 46.28(16.81-127.42) | 46.2(165.85) | 43.38(18.59) | 5.44(4.1) |
| Ketosis | 4 | 81.66(28.96-230.31) | 81.54(284.7) | 73.06(30.68) | 6.19(4.82) |
| Injection Site Discolouration | 3 | 7.82(2.51-24.43) | 7.82(17.64) | 7.74(2.99) | 2.95(1.5) |
| Staring | 3 | 5.56(1.79-17.34) | 5.56(11.13) | 5.52(2.13) | 2.47(1.01) |
| Diabetic Neuropathy | 3 | 693.88(139.98-3439.62) | 693.05(1036.58) | 347.02(90.92) | 8.44(6.63) |
| Abscess | 3 | 5.38(1.73-16.76) | 5.37(10.6) | 5.34(2.06) | 2.42(0.97) |
| Hyperthyroidism | 3 | 6.8(2.18-21.22) | 6.79(14.68) | 6.74(2.6) | 2.75(1.3) |
| Hypoglycaemic Coma | 3 | 47.31(14.68-152.46) | 47.25(127.16) | 44.3(16.64) | 5.47(3.97) |
| Hyperventilation | 3 | 5.7(1.83-17.77) | 5.7(11.52) | 5.66(2.19) | 2.5(1.05) |

Abbreviation: ROR, reporting odds ratio; PRR, proportional reporting ratio; EBGM, empirical Bayesian geometric mean; EBGM05, the lower limit of the 95% CI of EBGM; IC, information component; IC025, the lower limit of the 95% CI of the IC; CI, confidence interval; PT, preferred term.

Supplementary Table 4:

Top 50 most frequent ADEs for insulin glargine at the PT level in patients aged 18 to 65 from FAERS data

| **PT** | **Case numbers** | **ROR(95%CI)** | **PRR(χ^2^)** | **EBGM**  **(EBGM05)** | **IC(IC025)** |
| --- | --- | --- | --- | --- | --- |
| Visual Impairment | 1401 | 12.75(12.08-13.46) | 12.5(14267.91) | 12.05(11.52) | 3.59(3.51) |
| Hypoglycaemia | 1272 | 28.95(27.32-30.68) | 28.41(30802.03) | 26.08(24.85) | 4.7(4.62) |
| Injection Site Pain | 978 | 2.73(2.56-2.9) | 2.7(1043.84) | 2.69(2.55) | 1.43(1.33) |
| Cerebrovascular Accident | 553 | 4.03(3.7-4.38) | 4(1231.9) | 3.96(3.69) | 1.99(1.86) |
| Diabetes Mellitus Inadequate Control | 473 | 27.92(25.4-30.68) | 27.73(11174.7) | 25.5(23.56) | 4.67(4.53) |
| Vision Blurred | 383 | 2.38(2.16-2.64) | 2.38(303.71) | 2.37(2.17) | 1.24(1.09) |
| Injection Site Haemorrhage | 318 | 3.19(2.86-3.57) | 3.18(472.17) | 3.16(2.88) | 1.66(1.5) |
| Cataract | 314 | 9.07(8.1-10.15) | 9.03(2179.38) | 8.8(8.01) | 3.14(2.97) |
| Blindness | 308 | 9.32(8.32-10.44) | 9.28(2210.73) | 9.04(8.22) | 3.18(3.01) |
| Diabetic Ketoacidosis | 269 | 7.91(7.01-8.93) | 7.88(1576.86) | 7.71(6.97) | 2.95(2.77) |
| Injection Site Bruising | 255 | 2.49(2.2-2.82) | 2.49(225.52) | 2.48(2.23) | 1.31(1.13) |
| Visual Acuity Reduced | 243 | 7.48(6.59-8.5) | 7.46(1327.36) | 7.3(6.57) | 2.87(2.68) |
| Neuropathy Peripheral | 241 | 2.7(2.38-3.07) | 2.7(255.71) | 2.68(2.41) | 1.42(1.24) |
| Cardiac Disorder | 215 | 3.19(2.79-3.65) | 3.19(319.72) | 3.16(2.83) | 1.66(1.46) |
| Eye Disorder | 189 | 6.94(6.01-8.02) | 6.93(937.52) | 6.8(6.02) | 2.76(2.55) |
| Renal Disorder | 178 | 5.04(4.35-5.85) | 5.03(565.83) | 4.97(4.39) | 2.31(2.09) |
| Eye Haemorrhage | 141 | 13.25(11.2-15.69) | 13.23(1527.98) | 12.72(11.05) | 3.67(3.42) |
| Ketoacidosis | 112 | 15.04(12.44-18.19) | 15.02(1396.87) | 14.36(12.25) | 3.84(3.57) |
| Hypoglycaemic Coma | 105 | 46.98(38.25-57.7) | 46.91(4089.85) | 40.8(34.35) | 5.35(5.05) |
| Glaucoma | 102 | 8.07(6.63-9.83) | 8.06(614.88) | 7.88(6.68) | 2.98(2.69) |
| Retinopathy | 86 | 19.58(15.74-24.35) | 19.55(1422.98) | 18.44(15.36) | 4.2(3.89) |
| Hypoacusis | 85 | 4.46(3.6-5.53) | 4.46(224.8) | 4.41(3.68) | 2.14(1.83) |
| Localised Infection | 83 | 3.31(2.67-4.11) | 3.31(132.3) | 3.28(2.74) | 1.72(1.4) |
| Hypoglycaemic Unconsciousness | 72 | 38.71(30.29-49.47) | 38.67(2345.13) | 34.43(28.05) | 5.11(4.75) |
| Blindness Unilateral | 66 | 5.62(4.41-7.17) | 5.62(245.94) | 5.53(4.51) | 2.47(2.11) |
| Retinal Detachment | 60 | 6.29(4.87-8.13) | 6.29(261.45) | 6.18(4.99) | 2.63(2.25) |
| Hypoglycaemic Seizure | 57 | 67.57(50.71-90.02) | 67.51(3059.07) | 55.47(43.63) | 5.79(5.38) |
| Diabetic Neuropathy | 53 | 9.15(6.96-12.03) | 9.14(373.32) | 8.91(7.09) | 3.16(2.76) |
| Injection Site Discolouration | 51 | 2.91(2.21-3.83) | 2.91(63.28) | 2.89(2.29) | 1.53(1.13) |
| Thyroid Disorder | 51 | 3.4(2.58-4.48) | 3.4(85.28) | 3.37(2.67) | 1.75(1.35) |
| Diabetic Metabolic Decompensation | 42 | 28.16(20.53-38.63) | 28.14(1006.84) | 25.86(19.85) | 4.69(4.23) |
| Retinal Haemorrhage | 40 | 6.44(4.71-8.8) | 6.43(179.77) | 6.32(4.86) | 2.66(2.2) |
| Injection Site Injury | 35 | 6.35(4.54-8.88) | 6.35(154.47) | 6.24(4.71) | 2.64(2.16) |
| Injection Site Extravasation | 35 | 2.67(1.92-3.73) | 2.67(36.3) | 2.66(2.01) | 1.41(0.93) |
| Insulin Resistance | 33 | 14.99(10.57-21.26) | 14.98(410.53) | 14.33(10.7) | 3.84(3.33) |
| Gangrene | 32 | 4.15(2.93-5.88) | 4.15(75.4) | 4.1(3.07) | 2.04(1.53) |
| Impaired Gastric Emptying | 32 | 2.94(2.08-4.16) | 2.94(40.55) | 2.92(2.18) | 1.55(1.04) |
| Diabetic Complication | 31 | 12.34(8.62-17.67) | 12.33(310.35) | 11.89(8.81) | 3.57(3.05) |
| Macular Degeneration | 28 | 8.47(5.82-12.34) | 8.47(179.53) | 8.27(6.04) | 3.05(2.5) |
| Arterial Occlusive Disease | 23 | 2.88(1.91-4.35) | 2.88(28.02) | 2.87(2.03) | 1.52(0.93) |
| Diabetic Hyperglycaemic Coma | 22 | 73.09(45.9-116.39) | 73.07(1262.06) | 59.16(40.09) | 5.89(5.23) |
| Hypoglycaemia Unawareness | 20 | 36.39(22.89-57.85) | 36.38(614.89) | 32.61(22.13) | 5.03(4.36) |
| Shock Hypoglycaemic | 18 | 44.37(27.06-72.74) | 44.36(666.13) | 38.86(25.69) | 5.28(4.58) |
| Injection Site Scar | 17 | 5.55(3.44-8.97) | 5.55(62.28) | 5.47(3.66) | 2.45(1.76) |
| Lipodystrophy Acquired | 17 | 4.91(3.04-7.92) | 4.91(52.02) | 4.84(3.24) | 2.28(1.59) |
| Retinal Disorder | 15 | 6.53(3.92-10.89) | 6.53(68.77) | 6.41(4.18) | 2.68(1.95) |
| Multi-Organ Disorder | 15 | 5.92(3.55-9.87) | 5.92(60.2) | 5.83(3.8) | 2.54(1.81) |
| Neuropathic Arthropathy | 14 | 15.5(9.06-26.52) | 15.5(180.73) | 14.8(9.44) | 3.89(3.12) |
| Ketosis | 13 | 12.49(7.17-21.76) | 12.49(132.04) | 12.04(7.57) | 3.59(2.8) |
| Pancreatitis Chronic | 13 | 4.64(2.68-8.03) | 4.64(36.58) | 4.59(2.9) | 2.2(1.42) |

Abbreviation: ROR, reporting odds ratio; PRR, proportional reporting ratio; EBGM, empirical Bayesian geometric mean; EBGM05, the lower limit of the 95% CI of EBGM; IC, information component; IC025, the lower limit of the 95% CI of the IC; CI, confidence interval; PT, preferred term.

Supplementary Table 5:

Top 50 most frequent ADEs for insulin glargine at the PT level in patients aged over 65 from FAERS data

| PT | Case numbers | ROR(95%CI) | PRR(χ^2^) | EBGM  (EBGM05) | IC(IC025) |  |
| --- | --- | --- | --- | --- | --- | --- |
| Visual Impairment | 2217 | 17.14(16.39-17.92) | 16.66(29206.67) | 14.99(14.44) | 3.91(3.84) |  |
| Hypoglycaemia | 1133 | 13.3(12.5-14.14) | 13.11(11599.18) | 12.07(11.47) | 3.59(3.5) |  |
| Injection Site Pain | 1111 | 4.91(4.63-5.22) | 4.86(3298.13) | 4.73(4.49) | 2.24(2.15) |  |
| Cerebrovascular Accident | 775 | 2.64(2.46-2.84) | 2.63(770.12) | 2.6(2.45) | 1.38(1.27) |  |
| Hyperglycaemia | 749 | 14.15(13.12-15.26) | 14.02(8235.76) | 12.83(12.05) | 3.68(3.57) |  |
| Cataract | 695 | 6.15(5.7-6.64) | 6.1(2845.6) | 5.89(5.52) | 2.56(2.45) |  |
| Hypoacusis | 625 | 8.85(8.16-9.6) | 8.78(4059.18) | 8.32(7.78) | 3.06(2.94) |  |
| Injection Site Haemorrhage | 475 | 5.52(5.03-6.05) | 5.49(1678.7) | 5.32(4.92) | 2.41(2.28) |  |
| Memory Impairment | 452 | 2.86(2.6-3.14) | 2.85(531.97) | 2.81(2.6) | 1.49(1.35) |  |
| Hyperhidrosis | 370 | 2.53(2.28-2.81) | 2.52(335.11) | 2.5(2.29) | 1.32(1.17) |  |
| Visual Acuity Reduced | 353 | 6.55(5.89-7.29) | 6.53(1578.91) | 6.28(5.74) | 2.65(2.49) |  |
| Injection Site Bruising | 321 | 4.49(4.02-5.02) | 4.47(839.92) | 4.37(3.98) | 2.13(1.96) |  |
| Cardiac Disorder | 319 | 2.3(2.06-2.57) | 2.3(230.55) | 2.28(2.08) | 1.19(1.02) |  |
| Eye Disorder | 309 | 7.22(6.44-8.1) | 7.2(1569.23) | 6.89(6.26) | 2.79(2.62) |  |
| Blindness | 281 | 6.6(5.85-7.44) | 6.58(1269.75) | 6.33(5.72) | 2.66(2.49) |  |
| Renal Disorder | 247 | 3.4(2.99-3.85) | 3.39(406.37) | 3.33(3) | 1.74(1.55) |  |
| Dementia | 217 | 3.85(3.36-4.41) | 3.84(444.26) | 3.77(3.36) | 1.91(1.71) |  |
| Macular Degeneration | 162 | 7.26(6.2-8.5) | 7.25(829.46) | 6.94(6.08) | 2.79(2.56) |  |
| Glaucoma | 160 | 5.06(4.32-5.93) | 5.05(502.39) | 4.91(4.3) | 2.3(2.06) |  |
| Blindness Unilateral | 117 | 6.55(5.44-7.89) | 6.55(525.2) | 6.3(5.39) | 2.65(2.38) |  |
| Eye Haemorrhage | 104 | 4.92(4.04-5.98) | 4.91(313.24) | 4.78(4.06) | 2.26(1.97) |  |
| Injection Site Mass | 100 | 3.08(2.53-3.76) | 3.08(137.34) | 3.03(2.57) | 1.6(1.31) |  |
| Hypoglycaemic Coma | 78 | 11.5(9.13-14.49) | 11.49(690.06) | 10.69(8.81) | 3.42(3.08) |  |
| Hypoglycaemic Unconsciousness | 74 | 22.66(17.72-28.97) | 22.63(1316.58) | 19.61(15.97) | 4.29(3.94) |  |
| Injection Site Discolouration | 71 | 6.08(4.79-7.71) | 6.07(288.27) | 5.86(4.8) | 2.55(2.2) |  |
| Diabetic Retinopathy | 62 | 18.26(14.01-23.79) | 18.24(893.64) | 16.25(13.02) | 4.02(3.64) |  |
| Hunger | 52 | 5.01(3.8-6.61) | 5.01(161.24) | 4.87(3.87) | 2.28(1.88) |  |
| Diabetic Foot | 45 | 10.19(7.53-13.8) | 10.19(347.54) | 9.56(7.42) | 3.26(2.82) |  |
| Injection Site Extravasation | 45 | 3.78(2.81-5.09) | 3.78(89.72) | 3.71(2.9) | 1.89(1.46) |  |
| Retinopathy | 43 | 11.7(8.57-15.97) | 11.69(387.89) | 10.86(8.37) | 3.44(2.99) |  |
| Injection Site Injury | 41 | 10.65(7.75-14.64) | 10.65(333.04) | 9.96(7.64) | 3.32(2.86) |  |
| Pancreatic Disorder | 39 | 7.15(5.18-9.87) | 7.15(196.18) | 6.85(5.23) | 2.78(2.31) |  |
| Diabetic Coma | 39 | 16.95(12.16-23.64) | 16.95(521.79) | 15.22(11.52) | 3.93(3.45) |  |
| Diabetic Neuropathy | 37 | 6.02(4.33-8.36) | 6.01(148.31) | 5.81(4.41) | 2.54(2.06) |  |
| Gangrene | 34 | 3.31(2.35-4.65) | 3.3(53.39) | 3.25(2.45) | 1.7(1.21) |  |
| Injection Site Induration | 33 | 3.47(2.45-4.9) | 3.47(56.5) | 3.41(2.55) | 1.77(1.27) |  |
| Injection Site Discomfort | 30 | 3.35(2.33-4.81) | 3.35(48.3) | 3.29(2.43) | 1.72(1.2) |  |
| Retinal Disorder | 27 | 7.59(5.16-11.19) | 7.59(146.58) | 7.25(5.24) | 2.86(2.3) |  |
| Ketoacidosis | 25 | 3.8(2.56-5.66) | 3.8(50.27) | 3.73(2.67) | 1.9(1.32) |  |
| Insulin Resistance | 24 | 18.2(11.89-27.85) | 18.19(344.94) | 16.21(11.35) | 4.02(3.41) |  |
| Diabetic Complication | 23 | 7.31(4.81-11.12) | 7.31(118.98) | 6.99(4.92) | 2.81(2.2) |  |
| Deafness Unilateral | 22 | 2.98(1.95-4.55) | 2.98(28.32) | 2.94(2.06) | 1.55(0.95) |  |
| Injection Site Scar | 20 | 14.76(9.31-23.41) | 14.76(231.99) | 13.44(9.14) | 3.75(3.09) |  |
| Throat Clearing | 20 | 5.68(3.63-8.89) | 5.68(74.13) | 5.5(3.78) | 2.46(1.82) |  |
| Reading Disorder | 18 | 7.11(4.43-11.42) | 7.11(89.96) | 6.82(4.59) | 2.77(2.09) |  |
| Diabetic Metabolic Decompensation | 18 | 6.9(4.3-11.07) | 6.9(86.48) | 6.62(4.45) | 2.73(2.05) |  |
| Hypoglycaemic Seizure | 15 | 23.51(13.6-40.63) | 23.51(276.62) | 20.26(12.82) | 4.34(3.57) |  |
| Shock Hypoglycaemic | 15 | 16.22(9.5-27.69) | 16.22(191.88) | 14.63(9.35) | 3.87(3.11) |  |
| Diabetic Hyperglycaemic Coma | 9 | 24.14(11.9-48.99) | 24.14(170.18) | 20.73(11.46) | 4.37(3.39) |  |
| Hypoglycaemic Encephalopathy | 9 | 8.84(4.51-17.34) | 8.84(58.85) | 8.37(4.76) | 3.07(2.12) |  |

Abbreviation: ROR, reporting odds ratio; PRR, proportional reporting ratio; EBGM, empirical Bayesian geometric mean; EBGM05, the lower limit of the 95% CI of EBGM; IC, information component; IC025, the lower limit of the 95% CI of the IC; CI, confidence interval; PT, preferred term.

Supplementary Table 6. ADEs characteristics across different countries

| **United States** | | **Brazil** | | **Egypt** | |
| --- | --- | --- | --- | --- | --- |
| PT | Number | PT | Number | PT | Number |
| Visual impairment | 5,244 | Hyperglycaemia | 378 | Death | 206 |
| Injection site pain | 3,207 | Hypoglycaemia | 253 | Hyperglycaemia | 93 |
| Hypoglycaemia | 1,679 | Visual impairment | 124 | Condition aggravated | 61 |
| Cerebrovascular accident | 1,560 | Cerebrovascular accident | 109 | Cerebrovascular accident | 39 |
| Cataract | 1,281 | Injection site pain | 101 | Liver disorder | 39 |
| Hypoacusis | 1,176 | Blindness | 83 | Cardiac disorder | 38 |
| Diabetes mellitus inadequate control | 1,151 | Vision blurred | 81 | Renal failure | 32 |
| Injection site haemorrhage | 1,142 | Cataract | 75 | Hypoglycaemia | 28 |
| Memory impairment | 1,002 | Tremor | 74 | Hepatitis c | 26 |
| Injection site bruising | 935 | Hyperhidrosis | 71 | Diabetic hyperglycaemic coma | 24 |
| Hyperglycaemia | 882 | Renal failure | 61 | Diabetic ketoacidosis | 21 |
| Blindness | 808 | Renal disorder | 60 | Coma | 20 |
| Injury associated with device | 781 | Syncope | 54 | Myocardial infarction | 19 |
| Visual acuity reduced | 697 | Cardiac disorder | 52 | Ketoacidosis | 17 |
| Eye disorder | 678 | Infarction | 51 | Renal disorder | 17 |
| Cardiac disorder | 601 | Dementia alzheimer's type | 48 | Pancreatic carcinoma | 15 |
| Arthritis | 495 | Eye haemorrhage | 46 | Renal impairment | 15 |
| Renal disorder | 488 | Diabetes mellitus inadequate control | 45 | Neoplasm malignant | 13 |
| Dementia | 388 | Visual acuity reduced | 45 | Cardiac arrest | 13 |
| Macular degeneration | 337 | Glaucoma | 45 | Thrombosis | 12 |

**Supplementary Table 7. ADEs characteristics at different drug dosages**

| **10IU** | | **20IU** | | **30IU** | | **40IU** | |
| --- | --- | --- | --- | --- | --- | --- | --- |
| **PT** | **Number** | **PT** | **Number** | **PT** | **Number** | **PT** | **Number** |
| Visual impairment | 54 | Visual impairment | 105 | Visual impairment | 120 | Visual impairment | 107 |
| Drug ineffective | 48 | Injection site pain | 67 | Injection site pain | 74 | Malaise | 50 |
| Injection site pain | 48 | Drug ineffective | 65 | Malaise | 54 | Cataract | 50 |
| Malaise | 40 | Malaise | 56 | Cerebrovascular accident | 52 | Injection site pain | 40 |
| Feeling abnormal | 35 | Hypoglycaemia | 44 | Drug ineffective | 46 | Memory impairment | 35 |
| Dizziness | 32 | Cataract | 43 | Visual acuity reduced | 46 | Drug ineffective | 34 |
| Hypoglycaemia | 31 | Dizziness | 40 | Hypoglycaemia | 41 | Visual acuity reduced | 31 |
| Hyperglycaemia | 28 | Cerebrovascular accident | 40 | Cataract | 30 | Cerebrovascular accident | 31 |
| Condition aggravated | 28 | Hyperglycaemia | 35 | Tremor | 30 | Condition aggravated | 28 |
| Hyperhidrosis | 27 | Visual acuity reduced | 34 | Dizziness | 29 | Hyperglycaemia | 26 |
| Vision blurred | 27 | Vision blurred | 31 | Dyspnoea | 28 | Dyspnoea | 25 |
| Injection site haemorrhage | 25 | Feeling abnormal | 30 | Vision blurred | 28 | Myocardial infarction | 25 |
| Asthenia | 24 | Hypoacusis | 27 | Condition aggravated | 28 | Dizziness | 22 |
| Injection site bruising | 23 | Dyspnoea | 26 | Myocardial infarction | 27 | Hyperhidrosis | 22 |
| Headache | 23 | Death | 26 | Feeling abnormal | 25 | Gait disturbance | 21 |
| Death | 22 | Injection site haemorrhage | 25 | Hyperhidrosis | 24 | Headache | 21 |
| Fatigue | 22 | Condition aggravated | 25 | Blindness | 24 | Pneumonia | 21 |
| Tremor | 22 | Headache | 24 | Gait disturbance | 24 | Feeling abnormal | 21 |
| Rash | 22 | Injection site bruising | 24 | Nausea | 23 | Hypoglycaemia | 20 |
| Hypoacusis | 22 | Myocardial infarction | 23 | Hyperglycaemia | 23 | Cardiac disorder | 18 |

Supplementary Table 8:

ADEs for insulin glargine excluding common medication co-usage at the PT level from FAERS data

| PT | Case numbers | ROR(95%CI) | PRR(χ^2^) | EBGM  (EBGM05) | IC(IC025) |
| --- | --- | --- | --- | --- | --- |
| Visual impairment* | 5,522 | 14.27 ( 13.88 - 14.67 ) | 13.92 ( 62891.13 ) | 13.25 ( 12.95 ) | 3.73 ( 3.69 ) |
| Hypoglycaemia | 3,420 | 21.77 ( 21.02 - 22.55 ) | 21.43 ( 61466.31 ) | 19.84 ( 19.26 ) | 4.31 ( 4.26 ) |
| Injection site pain | 3,292 | 3.37 ( 3.26 - 3.49 ) | 3.33 ( 5331.98 ) | 3.3 ( 3.21 ) | 1.72 ( 1.67 ) |
| Hyperglycaemia | 2,272 | 19.12 ( 18.32 - 19.96 ) | 18.93 ( 35908.25 ) | 17.68 ( 17.05 ) | 4.14 ( 4.08 ) |
| Cerebrovascular accident | 1,926 | 3.2 ( 3.06 - 3.34 ) | 3.18 ( 2845.28 ) | 3.15 ( 3.03 ) | 1.66 ( 1.59 ) |
| Diabetes mellitus inadequate control | 1,480 | 26.33 ( 24.95 - 27.78 ) | 26.15 ( 32454.05 ) | 23.79 ( 22.75 ) | 4.57 ( 4.49 ) |
| Cataract | 1,402 | 7.17 ( 6.8 - 7.56 ) | 7.13 ( 7191.84 ) | 6.96 ( 6.66 ) | 2.8 ( 2.72 ) |
| Hypoacusis | 1,267 | 8.89 ( 8.4 - 9.4 ) | 8.84 ( 8517.64 ) | 8.57 ( 8.18 ) | 3.1 ( 3.02 ) |
| Injection site haemorrhage | 1,153 | 4.34 ( 4.09 - 4.6 ) | 4.32 ( 2894.97 ) | 4.26 ( 4.06 ) | 2.09 ( 2.01 ) |
| Memory impairment | 1,053 | 2.18 ( 2.05 - 2.32 ) | 2.18 ( 664.26 ) | 2.16 ( 2.06 ) | 1.11 ( 1.02 ) |
| Blindness | 948 | 6.99 ( 6.55 - 7.45 ) | 6.96 ( 4711.79 ) | 6.8 ( 6.44 ) | 2.77 ( 2.67 ) |
| Injection site bruising | 900 | 3.45 ( 3.23 - 3.68 ) | 3.43 ( 1534.24 ) | 3.4 ( 3.22 ) | 1.77 ( 1.67 ) |
| Injury associated with device | 796 | 12.56 ( 11.7 - 13.49 ) | 12.52 ( 8041.53 ) | 11.98 ( 11.28 ) | 3.58 ( 3.48 ) |
| Cardiac disorder | 748 | 2.25 ( 2.09 - 2.42 ) | 2.24 ( 512.48 ) | 2.23 ( 2.1 ) | 1.16 ( 1.05 ) |
| Visual acuity reduced | 744 | 6.09 ( 5.67 - 6.55 ) | 6.08 ( 3082.42 ) | 5.96 ( 5.6 ) | 2.57 ( 2.47 ) |
| Eye disorder | 701 | 6.34 ( 5.88 - 6.84 ) | 6.32 ( 3066.6 ) | 6.19 ( 5.82 ) | 2.63 ( 2.52 ) |
| Renal disorder | 593 | 3.66 ( 3.37 - 3.97 ) | 3.65 ( 1125.17 ) | 3.61 ( 3.37 ) | 1.85 ( 1.73 ) |
| Diabetic ketoacidosis | 590 | 7.19 ( 6.62 - 7.8 ) | 7.17 ( 3047.67 ) | 7 ( 6.54 ) | 2.81 ( 2.69 ) |
| Dementia | 425 | 4.64 ( 4.22 - 5.11 ) | 4.63 ( 1189.92 ) | 4.57 ( 4.22 ) | 2.19 ( 2.05 ) |
| Glaucoma | 353 | 5.32 ( 4.79 - 5.91 ) | 5.31 ( 1210.04 ) | 5.22 ( 4.78 ) | 2.38 ( 2.23 ) |
| Macular degeneration | 345 | 8.77 ( 7.88 - 9.77 ) | 8.76 ( 2293.26 ) | 8.5 ( 7.77 ) | 3.09 ( 2.93 ) |
| Eye haemorrhage | 341 | 7.32 ( 6.57 - 8.16 ) | 7.31 ( 1806.21 ) | 7.13 ( 6.52 ) | 2.83 ( 2.68 ) |
| Blindness unilateral | 299 | 6.26 ( 5.58 - 7.02 ) | 6.25 ( 1288.14 ) | 6.13 ( 5.57 ) | 2.62 ( 2.45 ) |
| Injection site mass | 292 | 2.34 ( 2.08 - 2.62 ) | 2.34 ( 221.36 ) | 2.32 ( 2.11 ) | 1.22 ( 1.05 ) |
| Diabetic retinopathy | 273 | 25.05 ( 22.12 - 28.36 ) | 25.01 ( 5727.2 ) | 22.85 ( 20.59 ) | 4.51 ( 4.33 ) |
| Ketoacidosis | 254 | 9.97 ( 8.8 - 11.31 ) | 9.96 ( 1970.5 ) | 9.62 ( 8.66 ) | 3.27 ( 3.08 ) |
| Hypoglycaemic coma | 218 | 23.13 ( 20.13 - 26.57 ) | 23.11 ( 4224.9 ) | 21.26 ( 18.93 ) | 4.41 ( 4.21 ) |
| Localised infection | 207 | 2.45 ( 2.14 - 2.81 ) | 2.45 ( 175.95 ) | 2.44 ( 2.17 ) | 1.28 ( 1.08 ) |
| Back disorder | 186 | 3.26 ( 2.82 - 3.77 ) | 3.26 ( 288.24 ) | 3.23 ( 2.87 ) | 1.69 ( 1.48 ) |
| Hypoglycaemic unconsciousness | 185 | 27.6 ( 23.72 - 32.13 ) | 27.58 ( 4273.07 ) | 24.97 ( 21.99 ) | 4.64 ( 4.42 ) |
| Dementia alzheimer's type | 179 | 5.59 ( 4.82 - 6.48 ) | 5.58 ( 659.21 ) | 5.49 ( 4.85 ) | 2.46 ( 2.24 ) |
| Retinopathy | 173 | 13.61 ( 11.68 - 15.86 ) | 13.6 ( 1916.22 ) | 12.95 ( 11.4 ) | 3.7 ( 3.47 ) |
| Injection site discolouration | 165 | 3.95 ( 3.38 - 4.6 ) | 3.94 ( 357.16 ) | 3.9 ( 3.43 ) | 1.96 ( 1.74 ) |
| Cold sweat | 148 | 2.39 ( 2.04 - 2.81 ) | 2.39 ( 118.85 ) | 2.38 ( 2.08 ) | 1.25 ( 1.01 ) |
| Diabetic neuropathy | 134 | 7.61 ( 6.41 - 9.04 ) | 7.61 ( 746.44 ) | 7.41 ( 6.42 ) | 2.89 ( 2.64 ) |
| Injection site injury | 134 | 8.69 ( 7.32 - 10.33 ) | 8.69 ( 881.28 ) | 8.43 ( 7.3 ) | 3.08 ( 2.82 ) |
| Hunger | 130 | 3.32 ( 2.79 - 3.95 ) | 3.32 ( 207.87 ) | 3.29 ( 2.84 ) | 1.72 ( 1.46 ) |
| Pancreatic disorder | 130 | 8.18 ( 6.87 - 9.74 ) | 8.17 ( 792.71 ) | 7.95 ( 6.87 ) | 2.99 ( 2.73 ) |
| Retinal detachment | 126 | 4.03 ( 3.38 - 4.81 ) | 4.03 ( 282.88 ) | 3.98 ( 3.44 ) | 1.99 ( 1.74 ) |
| Diabetic coma | 125 | 12.4 ( 10.36 - 14.84 ) | 12.4 ( 1248.56 ) | 11.86 ( 10.21 ) | 3.57 ( 3.31 ) |
| Injection site extravasation | 121 | 2.59 ( 2.17 - 3.1 ) | 2.59 ( 117.11 ) | 2.58 ( 2.22 ) | 1.36 ( 1.1 ) |
| Diabetic foot | 117 | 10.88 ( 9.04 - 13.09 ) | 10.87 ( 1005.37 ) | 10.46 ( 8.96 ) | 3.39 ( 3.12 ) |
| Frustration tolerance decreased | 116 | 4.35 ( 3.62 - 5.23 ) | 4.35 ( 294.37 ) | 4.29 ( 3.68 ) | 2.1 ( 1.83 ) |
| Hypoglycaemic seizure | 114 | 39.92 ( 32.76 - 48.64 ) | 39.9 ( 3734.05 ) | 34.6 ( 29.32 ) | 5.11 ( 4.83 ) |
| Diabetic complication | 106 | 11.63 ( 9.57 - 14.13 ) | 11.63 ( 984.26 ) | 11.16 ( 9.48 ) | 3.48 ( 3.19 ) |
| Retinal haemorrhage | 104 | 4.23 ( 3.49 - 5.14 ) | 4.23 ( 252.45 ) | 4.18 ( 3.55 ) | 2.06 ( 1.78 ) |
| Diabetic metabolic decompensation | 103 | 23.09 ( 18.87 - 28.25 ) | 23.08 ( 1993.24 ) | 21.23 ( 17.93 ) | 4.41 ( 4.11 ) |
| Injection site discomfort | 84 | 2.57 ( 2.07 - 3.18 ) | 2.57 ( 79.54 ) | 2.55 ( 2.13 ) | 1.35 ( 1.04 ) |
| Gangrene | 74 | 3.4 ( 2.7 - 4.28 ) | 3.4 ( 123.59 ) | 3.37 ( 2.78 ) | 1.75 ( 1.42 ) |
| Insulin resistance | 73 | 10.73 ( 8.49 - 13.56 ) | 10.73 ( 617.57 ) | 10.33 ( 8.49 ) | 3.37 ( 3.03 ) |
| Infarction | 68 | 2.66 ( 2.1 - 3.38 ) | 2.66 ( 69.96 ) | 2.65 ( 2.17 ) | 1.4 ( 1.06 ) |
| Retinal disorder | 61 | 6.78 ( 5.26 - 8.74 ) | 6.78 ( 292.5 ) | 6.63 ( 5.36 ) | 2.73 ( 2.36 ) |
| Injection site scar | 56 | 5.99 ( 4.59 - 7.8 ) | 5.99 ( 227.21 ) | 5.87 ( 4.7 ) | 2.55 ( 2.17 ) |
| Shock hypoglycaemic | 48 | 30.64 ( 22.71 - 41.34 ) | 30.63 ( 1227.13 ) | 27.43 ( 21.35 ) | 4.78 ( 4.34 ) |
| Diabetic hyperglycaemic coma | 45 | 44.96 ( 32.74 - 61.73 ) | 44.95 ( 1641.66 ) | 38.31 ( 29.38 ) | 5.26 ( 4.8 ) |
| Throat clearing | 42 | 3.48 ( 2.56 - 4.72 ) | 3.48 ( 73.13 ) | 3.44 ( 2.67 ) | 1.78 ( 1.34 ) |
| Injection site atrophy | 41 | 7.04 ( 5.16 - 9.6 ) | 7.03 ( 206.48 ) | 6.87 ( 5.3 ) | 2.78 ( 2.33 ) |
| Diabetic nephropathy | 39 | 4.88 ( 3.55 - 6.7 ) | 4.88 ( 118 ) | 4.81 ( 3.69 ) | 2.26 ( 1.8 ) |
| Hypoglycaemia neonatal | 38 | 6.81 ( 4.94 - 9.4 ) | 6.81 ( 183.44 ) | 6.66 ( 5.08 ) | 2.74 ( 2.27 ) |
| Lipodystrophy acquired | 33 | 4.29 ( 3.04 - 6.05 ) | 4.29 ( 81.87 ) | 4.23 ( 3.18 ) | 2.08 ( 1.58 ) |
| Diabetic eye disease | 33 | 24.97 ( 17.46 - 35.71 ) | 24.97 ( 691 ) | 22.81 ( 16.91 ) | 4.51 ( 3.99 ) |
| Reading disorder | 31 | 3.84 ( 2.69 - 5.48 ) | 3.84 ( 64.14 ) | 3.8 ( 2.82 ) | 1.93 ( 1.41 ) |
| Hypoglycaemia unawareness | 30 | 23.99 ( 16.5 - 34.89 ) | 23.99 ( 603.68 ) | 22 ( 16.08 ) | 4.46 ( 3.92 ) |
| Lipohypertrophy | 29 | 13.75 ( 9.46 - 19.98 ) | 13.75 ( 325.16 ) | 13.09 ( 9.58 ) | 3.71 ( 3.17 ) |
| Ketosis | 27 | 8.92 ( 6.08 - 13.09 ) | 8.92 ( 183.37 ) | 8.65 ( 6.27 ) | 3.11 ( 2.56 ) |
| Myopia | 27 | 3.24 ( 2.22 - 4.74 ) | 3.24 ( 41.32 ) | 3.21 ( 2.34 ) | 1.68 ( 1.14 ) |
| Injection site hypersensitivity | 26 | 2.93 ( 1.99 - 4.31 ) | 2.93 ( 32.66 ) | 2.91 ( 2.1 ) | 1.54 ( 0.98 ) |
| Neuropathic arthropathy | 24 | 10.59 ( 7.04 - 15.93 ) | 10.58 ( 199.92 ) | 10.2 ( 7.25 ) | 3.35 ( 2.76 ) |
| Hypoglycaemic encephalopathy | 24 | 19.01 ( 12.56 - 28.79 ) | 19.01 ( 380.9 ) | 17.75 ( 12.55 ) | 4.15 ( 3.55 ) |
| Dyslexia | 23 | 5.98 ( 3.95 - 9.04 ) | 5.98 ( 93.18 ) | 5.86 ( 4.15 ) | 2.55 ( 1.96 ) |
| Vascular occlusion | 23 | 3.08 ( 2.04 - 4.64 ) | 3.08 ( 31.89 ) | 3.05 ( 2.16 ) | 1.61 ( 1.02 ) |
| Pancreatic neoplasm | 21 | 5.02 ( 3.26 - 7.73 ) | 5.02 ( 66.21 ) | 4.94 ( 3.44 ) | 2.3 ( 1.68 ) |
| Fat tissue increased | 20 | 4.04 ( 2.6 - 6.28 ) | 4.04 ( 45.03 ) | 3.99 ( 2.76 ) | 2 ( 1.36 ) |
| Diabetic foot infection | 19 | 3.57 ( 2.27 - 5.62 ) | 3.57 ( 34.71 ) | 3.54 ( 2.42 ) | 1.82 ( 1.17 ) |
| Hypermetropia | 19 | 5.53 ( 3.51 - 8.72 ) | 5.53 ( 69.03 ) | 5.43 ( 3.72 ) | 2.44 ( 1.79 ) |
| Injection site hypertrophy | 19 | 20.52 ( 12.86 - 32.75 ) | 20.52 ( 326.29 ) | 19.05 ( 12.88 ) | 4.25 ( 3.58 ) |
| Amyloidosis | 18 | 3 ( 1.89 - 4.78 ) | 3 ( 23.73 ) | 2.98 ( 2.02 ) | 1.57 ( 0.91 ) |
| Dawn phenomenon | 17 | 55.08 ( 32.6 - 93.08 ) | 55.08 ( 741.1 ) | 45.4 ( 29.27 ) | 5.5 ( 4.76 ) |
| Colour blindness | 16 | 4.96 ( 3.02 - 8.13 ) | 4.96 ( 49.54 ) | 4.88 ( 3.22 ) | 2.29 ( 1.58 ) |
| Hyperinsulinaemic hypoglycaemia | 16 | 27.32 ( 16.31 - 45.76 ) | 27.32 ( 366.12 ) | 24.75 ( 16.08 ) | 4.63 ( 3.89 ) |
| Diabetic retinal oedema | 14 | 7.97 ( 4.68 - 13.57 ) | 7.97 ( 82.7 ) | 7.76 ( 4.97 ) | 2.96 ( 2.2 ) |
| Ketonuria | 13 | 4.52 ( 2.61 - 7.82 ) | 4.52 ( 35 ) | 4.46 ( 2.82 ) | 2.16 ( 1.38 ) |
| Starvation | 13 | 5.46 ( 3.15 - 9.45 ) | 5.46 ( 46.32 ) | 5.36 ( 3.39 ) | 2.42 ( 1.64 ) |
| Cutaneous amyloidosis | 12 | 48.91 ( 26.36 - 90.76 ) | 48.91 ( 471.85 ) | 41.14 ( 24.53 ) | 5.36 ( 4.5 ) |
| Injection site laceration | 11 | 4.89 ( 2.69 - 8.87 ) | 4.89 ( 33.35 ) | 4.81 ( 2.92 ) | 2.27 ( 1.43 ) |
| Coma hepatic | 10 | 3.47 ( 1.86 - 6.48 ) | 3.47 ( 17.36 ) | 3.44 ( 2.04 ) | 1.78 ( 0.91 ) |
| Diabetic blindness | 10 | 35.1 ( 18.11 - 68.01 ) | 35.1 ( 290.87 ) | 30.94 ( 17.79 ) | 4.95 ( 4.03 ) |
| Stomach mass | 10 | 3.73 ( 2 - 6.96 ) | 3.73 ( 19.67 ) | 3.69 ( 2.19 ) | 1.88 ( 1.01 ) |
| Hyperglycaemic unconsciousness | 9 | 20.68 ( 10.48 - 40.8 ) | 20.68 ( 155.77 ) | 19.19 ( 10.87 ) | 4.26 ( 3.31 ) |
| Brain stem stroke | 9 | 5.19 ( 2.68 - 10.05 ) | 5.19 ( 29.85 ) | 5.11 ( 2.94 ) | 2.35 ( 1.43 ) |
| Retinopathy proliferative | 9 | 13.38 ( 6.84 - 26.16 ) | 13.38 ( 97.9 ) | 12.76 ( 7.28 ) | 3.67 ( 2.74 ) |
| Acetonaemia | 8 | 9.86 ( 4.87 - 19.99 ) | 9.86 ( 61.31 ) | 9.53 ( 5.28 ) | 3.25 ( 2.27 ) |
| Insulin autoimmune syndrome | 8 | 8.42 ( 4.16 - 17.04 ) | 8.42 ( 50.65 ) | 8.18 ( 4.54 ) | 3.03 ( 2.05 ) |
| Diabetic gastroparesis | 8 | 5.88 ( 2.92 - 11.85 ) | 5.88 ( 31.64 ) | 5.77 ( 3.21 ) | 2.53 ( 1.55 ) |
| Drug effect faster than expected | 7 | 7.19 ( 3.39 - 15.24 ) | 7.19 ( 36.28 ) | 7.02 ( 3.74 ) | 2.81 ( 1.78 ) |
| Pyoderma | 7 | 4.05 ( 1.92 - 8.54 ) | 4.05 ( 15.81 ) | 4 ( 2.14 ) | 2 ( 0.97 ) |
| Lens disorder | 6 | 5.83 ( 2.6 - 13.1 ) | 5.83 ( 23.48 ) | 5.72 ( 2.91 ) | 2.52 ( 1.41 ) |
| Pancreas infection | 6 | 4.8 ( 2.14 - 10.76 ) | 4.8 ( 17.7 ) | 4.73 ( 2.4 ) | 2.24 ( 1.14 ) |
| Malignant neoplasm of eye* | 6 | 6.34 ( 2.82 - 14.26 ) | 6.34 ( 26.35 ) | 6.21 ( 3.15 ) | 2.64 ( 1.53 ) |
| Kidney malformation | 6 | 4.83 ( 2.15 - 10.83 ) | 4.83 ( 17.87 ) | 4.76 ( 2.42 ) | 2.25 ( 1.15 ) |
| Diabetic ketoacidotic hyperglycaemic coma | 6 | 9.13 ( 4.05 - 20.63 ) | 9.13 ( 41.94 ) | 8.85 ( 4.48 ) | 3.15 ( 2.03 ) |
| Decreased insulin requirement | 6 | 30.33 ( 13 - 70.73 ) | 30.32 ( 151.92 ) | 27.18 ( 13.38 ) | 4.76 ( 3.61 ) |
| Diabetic glaucoma | 6 | 63.18 ( 25.82 - 154.56 ) | 63.18 ( 293.72 ) | 50.74 ( 24 ) | 5.67 ( 4.46 ) |
| Diabetic ketosis | 6 | 6.07 ( 2.7 - 13.63 ) | 6.06 ( 24.78 ) | 5.95 ( 3.02 ) | 2.57 ( 1.47 ) |
| Cataract diabetic | 6 | 32.26 ( 13.79 - 75.46 ) | 32.26 ( 161.17 ) | 28.72 ( 14.11 ) | 4.84 ( 3.68 ) |
| Medullary thyroid cancer* | 5 | 5.03 ( 2.08 - 12.2 ) | 5.03 ( 15.85 ) | 4.96 ( 2.36 ) | 2.31 ( 1.12 ) |
| Acanthosis nigricans | 5 | 7.7 ( 3.16 - 18.76 ) | 7.7 ( 28.31 ) | 7.51 ( 3.57 ) | 2.91 ( 1.71 ) |
| Increased insulin requirement | 5 | 6.29 ( 2.59 - 15.27 ) | 6.29 ( 21.69 ) | 6.16 ( 2.93 ) | 2.62 ( 1.43 ) |
| Administration site bruise | 5 | 4.59 ( 1.9 - 11.13 ) | 4.59 ( 13.81 ) | 4.53 ( 2.16 ) | 2.18 ( 0.99 ) |
| Sodium retention | 5 | 6.83 ( 2.81 - 16.6 ) | 6.83 ( 24.23 ) | 6.68 ( 3.17 ) | 2.74 ( 1.54 ) |
| Cerebrovascular stenosis | 4 | 7.43 ( 2.75 - 20.09 ) | 7.43 ( 21.63 ) | 7.25 ( 3.15 ) | 2.86 ( 1.54 ) |
| Diabetic foetopathy | 4 | 16.85 ( 6.12 - 46.36 ) | 16.85 ( 55.9 ) | 15.86 ( 6.8 ) | 3.99 ( 2.65 ) |
| Postprandial hypoglycaemia | 4 | 7.43 ( 2.75 - 20.09 ) | 7.43 ( 21.63 ) | 7.25 ( 3.15 ) | 2.86 ( 1.54 ) |
| Erythema induratum | 4 | 8.71 ( 3.22 - 23.61 ) | 8.71 ( 26.4 ) | 8.46 ( 3.67 ) | 3.08 ( 1.76 ) |
| Benign pancreatic neoplasm* | 4 | 6.13 ( 2.27 - 16.52 ) | 6.13 ( 16.75 ) | 6 ( 2.62 ) | 2.59 ( 1.28 ) |
| Chronic disease | 4 | 5.05 ( 1.88 - 13.6 ) | 5.05 ( 12.75 ) | 4.97 ( 2.17 ) | 2.31 ( 1.01 ) |
| Diabetic wound | 4 | 16.3 ( 5.93 - 44.81 ) | 16.3 ( 53.98 ) | 15.38 ( 6.6 ) | 3.94 ( 2.6 ) |
| Diabetic hepatopathy | 4 | 101.08 ( 31.7 - 322.31 ) | 101.08 ( 283.12 ) | 72.49 ( 27.47 ) | 6.18 ( 4.69 ) |
| Kussmaul respiration | 4 | 5.21 ( 1.94 - 14.02 ) | 5.21 ( 13.33 ) | 5.13 ( 2.24 ) | 2.36 ( 1.05 ) |
| Somogyi phenomenon | 3 | 50.54 ( 14.63 - 174.59 ) | 50.54 ( 121.4 ) | 42.28 ( 14.99 ) | 5.4 ( 3.81 ) |
| Bone marrow tumour cell infiltration* | 3 | 6.59 ( 2.1 - 20.74 ) | 6.59 ( 13.87 ) | 6.45 ( 2.47 ) | 2.69 ( 1.22 ) |
| Injection site fibrosis | 3 | 7.09 ( 2.25 - 22.32 ) | 7.09 ( 15.25 ) | 6.92 ( 2.65 ) | 2.79 ( 1.32 ) |
| Congenital bladder anomaly | 3 | 5.83 ( 1.86 - 18.32 ) | 5.83 ( 11.74 ) | 5.72 ( 2.2 ) | 2.52 ( 1.05 ) |
| Abdominal fat apron | 3 | 9.36 ( 2.96 - 29.63 ) | 9.36 ( 21.6 ) | 9.06 ( 3.45 ) | 3.18 ( 1.7 ) |
| Vessel puncture site injury | 3 | 31.59 ( 9.51 - 104.91 ) | 31.59 ( 78.99 ) | 28.19 ( 10.33 ) | 4.82 ( 3.27 ) |
| Abnormal labour | 3 | 6.32 ( 2.01 - 19.87 ) | 6.32 ( 13.1 ) | 6.19 ( 2.37 ) | 2.63 ( 1.16 ) |

Abbreviation: ROR, reporting odds ratio; PRR, proportional reporting ratio; EBGM, empirical Bayesian geometric mean; EBGM05, the lower limit of the 95% CI of EBGM; IC, information component; IC025, the lower limit of the 95% CI of the IC; CI, confidence interval; PT, preferred term. * The asterisks indicate noteworthy and unexpected adverse events that are not listed in the drug’s label.
